# Psychological health and safety of criminal justice workers: a scoping review of strategies and supporting research

**DOI:** 10.1186/s40352-025-00320-0

**Published:** 2025-02-26

**Authors:** Christopher Canning, Tyler Szusecki, N. Zoe Hilton, Elnaz Moghimi, Ashley Melvin, Matthew Duquette, Jolene Wintermute, Nicole Adams

**Affiliations:** 1 Waypoint Centre for Mental Health Care, 500 Church Street, Penetanguishene, ON L9M 1G3 Canada; 2 Department of Psychiatry, University of Toronto, 250 College Street, Toronto, ON M5T 1R8 Canada; 3 Department of Psychiatry, Faculty of Health Sciences, Queen’s University, 99 University Avenue, Kingston, ON K7L 3N6 Canada

**Keywords:** Criminal justice system, Forensic mental health, Psychological health and safety, Workplace mental health, Social-Ecological Model

## Abstract

**Background:**

People working in the criminal justice system face substantial occupational stressors due to their roles involving high-risk situations, trauma exposure, heavy workloads, and responsibility for public safety. Consequently, they have a higher prevalence of mental health problems than the general population. Employees identifying as women, Two-Spirit, Lesbian, Gay, Bisexual, Transgender, Queer, Intersexual, Asexual, and all others (2SLGBTQIA+), or Black, Indigenous, and People of Color (BIPOC), may experience additional stressors due to discrimination, harassment, and systemic barriers to seeking and receiving support. Psychoeducational and psychosocial programs have shown mixed effectiveness for preventing or reducing occupational stress, emphasizing the urgent need for multi-level, comprehensive, system-wide approaches. This scoping review aimed to capture and consolidate recommendations from strategies, frameworks, and guidelines on supporting the psychological health of criminal justice workers.

**Results:**

The scoping review of 65 grey and 85 academic literature records presents recommendations aimed at improving the psychological health and safety of criminal justice system workers. Findings were mapped by occupational groups to the Social-Ecological Model and accounted for factors across the individual, interpersonal, institutional, and policy levels. The most common recommendation across all criminal justice occupational groups was workplace mental health training to reduce stigma, encourage help-seeking, prepare workers for traumatic incidents, and promote culturally responsive approaches. At the individual level, physical health, healthy lifestyle choices, and coping strategies were widely recommended. Interpersonal interventions, including peer support and models emphasizing wraparound care, were also recommended. Institutional factors such as fair workloads, safe working conditions, and harassment-free workplaces were emphasized. At the policy level, presumptive coverage policies and adequate funding for staffing needs were highlighted.

**Conclusion:**

This scoping review captured intersecting strategies and recommendations, consisting primarily of individual- and institutional-level supports and services. Fewer records discussed the need to address structural and policy considerations such as labor shortages, patchy mental health benefits, underfunding, and discrimination. The review highlights the need for shared responsibility across different levels, providing a framework for improving the psychological health and safety of criminal justice workers.

**Supplementary Information:**

The online version contains supplementary material available at 10.1186/s40352-025-00320-0.

Criminal justice workers primarily engage in law enforcement, corrections, judicial proceedings, forensic healthcare, and public safety. Numerous studies have highlighted the effect of occupational stressors on the mental health of employees in the criminal justice system (Hilton et al., 2021; Ricciardelli et al., 2023b). These stressors may be due to exposure to trauma, high-risk environments, heavy workloads, organizational challenges, and legal and ethical pressures inherent in these professions (Abeyta, 2021). There is a notable link between poor employee mental health and exposure to traumatic incidents such as workplace violence (Ricciardelli et al., 2018a; Rodrigues et al., 2021a, b). Moreover, organizational stressors such as resource shortages, workload demands, interpersonal relationship dynamics, and burdensome administrative duties can exacerbate burnout and psychological injuries following exposure to traumatic incidents (Cadieux et al., 2020; Ricciardelli et al., 2020b; Rodrigues et al., 2021a, b; Swenson et al., 2020). Documented impacts include depression, anxiety, post-traumatic stress, chronic pain, burnout, compassion fatigue, moral injury, sleep disorders, suicidal ideation, and substance use (Seto et al., 2020). Almost half of public safety personnel—including correctional workers and police officers—screen positive for clinically significant symptoms of at least one mental disorder (Carleton et al., 2021). Workplace stressors and barriers to seeking support are likely intensified for employees with marginalized identities (e.g., women, 2SLGBTQIA+, and BIPOC) because of discrimination, harassment, and stereotyping (Bastarache, 2021; Batton et al., 2019; Giwa et al., 2022; Martinez, 2019).

To support the mental health needs of workers, many organizations offer psychoeducational or psychosocial programs to mitigate adverse mental health outcomes and promote resilience (Papazoglou et al., 2021; Rodriguez et al., 2023). However, previous research findings on the effectiveness of such programs are mixed (Vanhove et al., 2016; Anderson et al., 2020). Increasingly, the emphasis is shifting away from standalone interventions at the individual level in favor of multi-level, wraparound strategies to support workers’ psychological health and safety (Jessiman-Perreault et al., 2021; Johnston et al., 2022). Supporting this holistic approach requires synthesis of current evidence-based practices across multiple spheres of influence on individual behavior and well-being.

This review captures and consolidates recommendations from strategies, frameworks, guidelines, and interventions in academic and gray literature to support the psychological health and safety of workers across all occupations in or related to the criminal justice system, including, for example, victim advocates, paralegals, and probation officers (see Supplemental Table 1: Definitions of Terminology).

Scoping reviews are advantageous to map research areas that have not been comprehensively reviewed (Arksey & O’Malley, 2005). As no prior research has consolidated recommendations for how best to support the psychological health and safety of criminal justice workers, a scoping review enabled us to examine the literature and map available evidence across different occupations of the criminal justice system (Munn et al., 2018). This review drew on the Social-Ecological Model (SEM) to describe the relationship between individual behaviors and practices, physical environments, social and structural factors, and health (Baral et al., 2013). The SEM helps to contextualize different factors involved in an individuals’ susceptibility to negative health outcomes, while also delineating actions to safeguard well-being across five levels: individual (e.g., knowledge, attitudes, behavior), interpersonal (peer/social support), institutional (e.g., regulations, policies), community (e.g. relationships between organizations/ institutions), and public policy (e.g. local, provincial/state, national/federal laws and regulations). In the current review, we used an adapted version of the SEM to map recommendations across four levels (Dahlberg & Krug, 2002). By systematically mapping recommendations rooted in existing practices, this knowledge synthesis provides a descriptive and narrative account of individual, interpersonal, institutional, and policy-level findings.

## Methods

The review followed the Joanna Briggs Institute (JBI) guidelines for scoping reviews and was guided by an unpublished a priori protocol, including the Preferred Reporting Items for Systematic Reviews and Meta-Analyses extension for Scoping Reviews (PRISMA-ScR) (Tricco et al., 2018). Prior to commencing the review, the protocol was refined through consultation with expert advisors from the Mental Health Commission of Canada (MHCC)’s National Advisory Group in Criminal Justice and Mental Health. Following feedback, supplementary searches targeted priority populations identified by the MHCC and other populations not covered in the original protocol. Specifically, records covered BIPOC, 2SLGBTQIA+, and women, in addition to victim advocates, NGOs, social workers, peer support workers, mobile crisis teams, probation and parole officers, and forensic administrative staff. The proposed Appraisal of Guidelines Research & Evaluation tool (AGREE-HS) was also rescinded as it was deemed not effective at appraising the quality of academic and gray literature documents containing recommendations from strategies, frameworks, and guidelines.

The research team finalized the research question through an iterative process and in consultation with MHCC’s expert advisors. The research question was: What are the strategies, frameworks, interventions, and guidelines within the literature supporting the psychological health and safety of people who work within, or whose work directly relates to, the criminal justice system? The sub-question was: What are the common themes and recommendations from this literature and what can we learn from them?

### Study eligibility

Eligible records were defined using a series of inclusion and exclusion criteria detailed in Table 1. Briefly, records needed to have data or recommendations from Canada, USA, Australia, New Zealand, UK, Norway, Sweden, Finland, or Denmark. The rationale to include these countries stems from similarities in their legal systems and criminal justice practices, ensuring consolidation of recommendations across similar contexts. Populations were considered if they worked within, or those whose work directly relates to, the criminal justice system – collectively referred to as criminal justice workers. For a complete list of included and excluded populations, see Supplemental Table 2.

**Table 1:** Inclusion and exclusion criteria for academic and gray literature

Criteria	Inclusion	Exclusion
**General Criteria**	**Dates**: Academic: 2017-present; Gray: 2007-present **Sources**: Relevant databases, NGO websites, and government websites from included jurisdictions	**Dates**: Academic: before 2017; Gray: before 2007 **Sources**: Infographics, brochures, webpages (other than organizational websites), conference abstracts, personal blogs, book and book chapters, dissertations and theses
**Populations**: Individuals who work within the criminal justice system (see full list in Supplemental Table 1)	Records that include individuals who work within the criminal justice system, even when consolidated into broader classifications such as public safety personnel (PSP)	Records that do not include individuals who work within the criminal justice system, or do not report on recommendations or evaluated strategies specific to these populations
**Concept**: Psychological health and safety in the workplace, defined as a workplace that promotes workers’ psychological well-being and actively works to prevent harm to worker psychological health, including in neglectful, reckless, or intentional ways (MHCC, 2013)	Records that address the psychological health and safety of the populations listed in Supplemental Table 1	Records that make recommendations about individual behavioral changes or interventions that support general well-being or mindfulness, but are not part of a broader organizational strategy to support the psychological health and safety of the populations included in the review
**Context**: Workplaces and occupations related to the criminal justice system and related fields	Records that include recommendations from specific frameworks, guidelines, strategies, or interventions that support psychological health and safety in these contexts	Records lacking recommendations that support psychological health and safety, or without a concentrated emphasis on mental health within these contexts

Records were included if they provided organizational or system-level recommendations or evaluations of specific frameworks, guidelines, or strategies that support the psychological health and safety of the eligible populations. Recommendations from studies investigating individual interventions (e.g., resilience training, mindfulness) were eligible if they were part of a broader organizational strategy and offered specific recommendations pertaining to the current context. The review focused on research articles, reviews, government and organizational reports, and policy documents. Infographics, brochures, webpages (other than organizational websites), conference abstracts, blogs, book and book chapters, studies that solely focused on intervention efficacy, and dissertations and theses were not included. The final search was executed on December 1, 2023.

### Search strategy

Academic records were identified and retrieved through MEDLINE, PsycINFO, Criminal Justice Abstracts, CINAHL, and Google Scholar, with searches limited to post-2017 to capture the most updated findings and developments in the field. Gray literature was identified through Health Systems Evidence, Social Systems Evidence, LexisNexis, Policy Commons, governmental and non-governmental websites from identified jurisdictions, Google Scholar, advanced Google searches, the MHCC database, and Non-Governmental Organization (NGO) searches. Gray literature was limited to post-2007, as governmental reports, policy documents, and organizational reports in this area are typically updated over longer timeframes.

While some gray literature sources included in this review predate the academic records, their inclusion was guided by enduring relevance to the field. These documents often outline foundational principles, frameworks, and interventions that continue to inform current practices and policies. The review prioritized gray literature that addressed ongoing issues in the criminal justice system to ensure recommendations remain current. Gray literature, such as policy reports and guidelines, provides practical insights and highlights emerging trends, while academic literature contributes methodologically robust, theory-driven evidence. Together, these sources bridge empirical evidence with actionable strategies to enhance the scope and applicability of the findings.

To ensure comprehensiveness, five key documents meeting the inclusion criteria were identified prior to conducting the searches. The presence of these records in the search results confirmed that the search strategy effectively captured the intended literature. Supplemental Table 3 contains a sample of the search strategy, as executed by the research team on July 28, 2023.

### Screening process

**Fig. 1:**
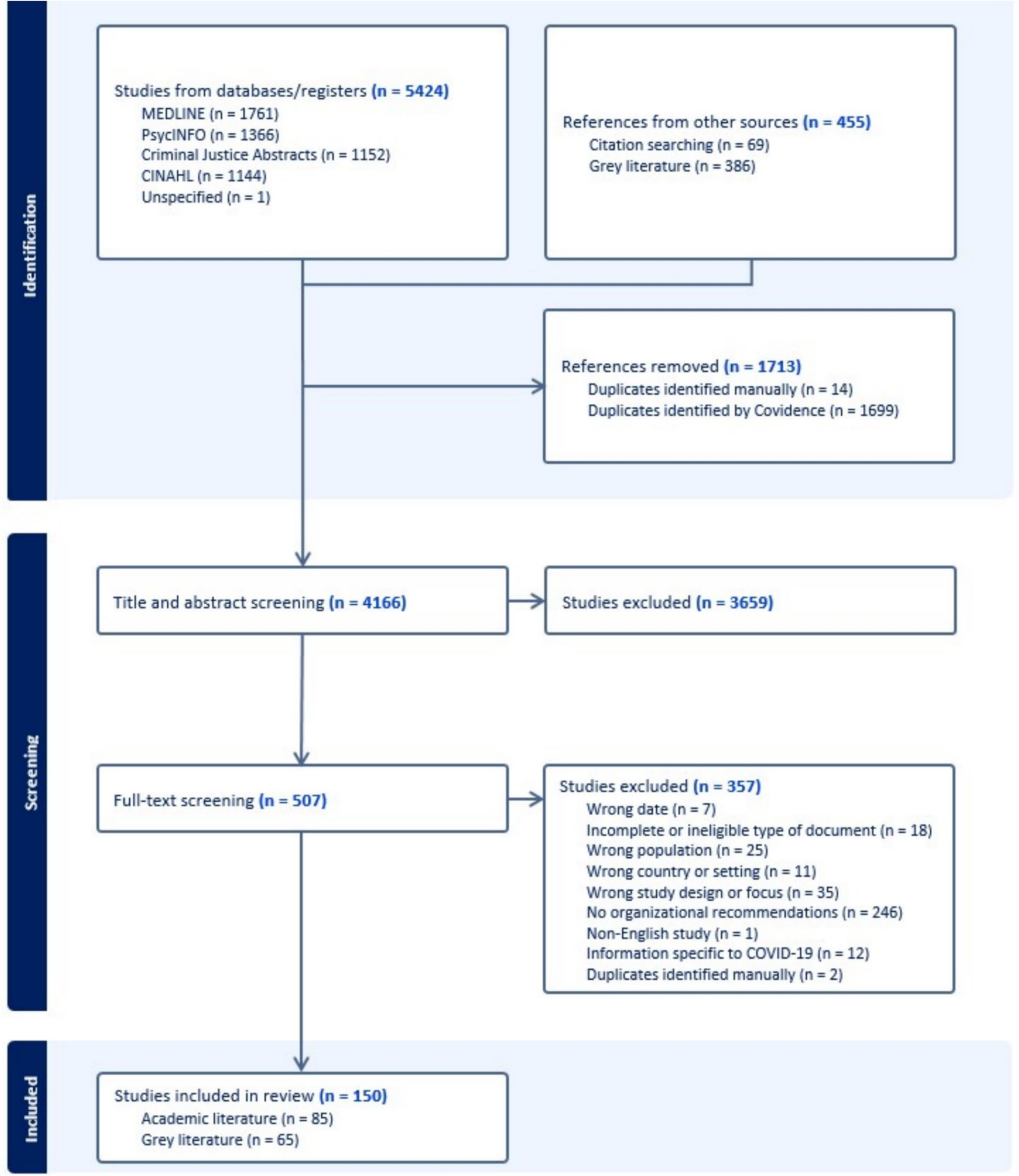
Summary of records included in the review

The search results were uploaded to Covidence, a web-based collaboration software platform that streamlines the production of reviews (Veritas Health Innovation, 2023). After removing 1,713 duplicates, a total of 4,166 records underwent abstract and title screening. Records were independently screened by two researchers and conflicts were resolved through discussions between the screeners and a third member of the research team. Unresolved conflicts were brought to the team’s attention for decision-making by consensus. From the 507 records eligible for full-text review, 357 were excluded due to limiting factors (e.g., wrong date, wrong population, no organizational recommendations). Altogether, 150 records (85 academic records and 65 Gy records) were identified for data extraction. A summary of the screening process can be found in Fig. 1.

### Data extraction

Data were charted using an extraction template developed by the research team under the following headings: Covidence number; citation; population(s), e.g., correctional officers, police and law enforcement personnel, lawyers, forensic and correctional nurses; priority populations, i.e., BIPOC, 2SLGBTQIA+, women, and individuals affected by mental illnesses or disabilities; jurisdictions, e.g., Canada, the USA, and Australia; reported gaps and limitations; recommendations; and stages of implementation, e.g., draft or concept, proposal, implemented but not evaluated, or audited or evaluated; and evaluation data.

Data extraction was conducted by two researchers per document for approximately half of the records. One researcher extracted data for the remaining documents and the first author reviewed and finalized the data. Charting conflicts were resolved through discussions with data extractors, or, if unresolved, by consensus with the full project team. Data were then imported to Microsoft Excel to clean, finalize, and map to the SEM levels.

### Mapping recommendations

The recommendations were categorized according to criminal justice professions specified in the data: (1) *Police and law enforcement personnel*; (2) *Correctional employees*; (3) *Legal staff and those directly involved in court proceedings* (e.g., lawyers, judges, paralegals, jurors); (4) *Mental health and health care providers* in forensic and correctional settings (e.g., nurses, psychologists, psychiatrists); (5) *Criminal justice system support workers* (e.g., probation and parole officers, victim advocates). Within each occupational category, common recommendations from the literature were synthesized into high-level themes. Themed recommendations were then charted and mapped to corresponding levels of the SEM (i.e., individual, interpersonal, institutional, and policy). For transparency, counts of recommendations per SEM level were based on the number of records discussing each themed recommendation. Each record was coded for all relevant themes and levels, and these counts were aggregated to produce the final numbers. The aggregated totals are captured below in charts under each occupational grouping. Mapping was conducted by two members of the research team and reviewed by all team members prior to finalization.

## Results

The review of 150 records drew from a diverse range of sources, including federal government agencies, unions, bar associations, and other professional associations (for a full list of included records, see Supplemental Table 4). Twenty populations comprising various occupations and roles were included, the most common being police and law enforcement (*n* = 98 records), correctional officers (*n* = 44 records), lawyers (*n* = 12 records), and forensic and correctional nurses (*n* = 10 records) (see Supplemental Fig. 1). Several populations appeared in a maximum of four records (i.e., mobile crisis support staff, psychometrists, forensic and correctional psychiatrists and psychologists). While 79% of records addressed a single occupational category (*n* = 119), 21% were cross-sectoral (*n* = 31).

Priority populations identified for the scoping review were women, 2SLGBTQIA+, BIPOC, and individuals affected by mental illness or disability. Approximately 78% of the records (*n* = 118) did not specify a priority population. Women were discussed in 15 records (10%), and individuals affected by mental illness or disability were discussed in 14 records (9%). BIPOC were addressed in four records (3%). Three records (2%) discussed the experiences of 2SLGBTQIA + communities.

Most of the records represented the USA or Canada (65 and 60 records, respectively). The UK was represented in 28 records and Australia was represented in 19 records. Additionally, New Zealand, Norway, and Sweden were represented with five, two, and one record(s) respectively, while Denmark and Finland each had one record.

### Overview of recommendations by SEM level

At the individual level, employees are responsible for behaviors that maintain their psychological health. This was referred to as self-care, wellness, well-being, healthy coping mechanisms, and healthy lifestyle habits in the records. Examples included nutrition, exercise, healthy relationships, mindfulness, and cognitive strategies like positive self-talk (Edwards et al., 2021; Stelnicki et al., 2021).

At the interpersonal level, the focus was on employees’ relationships with their colleagues, peers, and mentors. At this level, colleagues, peers, and supervisors are responsible for their actions toward others, and for mutual maintenance of a respectful, anti-stigma, anti-discrimination environment. The records emphasized the need for supportive peer-to-peer networks, along with healthy employee-manager communication. Peer support programs were widely recommended across all occupational groups. These programs are thought to reduce stigma, build resilience, and foster a sense of community within workplaces. While variations in implementation and effectiveness were noted, peer support was consistently emphasized as an important component of broader organizational strategies. The recurring emphasis on peer support is mapped throughout the results, reflecting its adaptability and relevance across all criminal justice professions.

The third level contained responsibilities within an organization and encompassed most of the strategies, interventions, and recommendations in this review. The findings highlighted a significant degree of accountability within this level, e.g., opportunities for organizational leaders to offer training, provide access to services and supports, and prevent and address post-traumatic stress injuries and other negative mental health outcomes in the workplace.

Finally, the policy level encompassed the wider social, economic, and political contexts. Recommendations here included providing adequate funding to organizations to support staffing needs and providing presumptive coverage for occupational stress injuries. At this level, provincial/territorial/state and federal governments are responsible for implementation.

### Recommendations by profession

#### Police and law enforcement personnel

Of the 150 total records, 98 (65%) discussed strategies, frameworks, and guidelines in support of police and law enforcement personnel psychological health and safety (including 49, 50% academic and 49, 50% gray records). Notably, the USA gray literature comprised nearly one-third of the total literature included for police and law enforcement (29 records; 30%). Most records (80, 82%) discussed police and law enforcement alone and the remaining 18 (18%) also concerned other public safety employees (e.g., correctional officers, lawyers, and probation and parole officers).

Recommendations in this category sought to prevent and improve outcomes related to occupational stress, psychological workplace injuries, suicidal ideation, and other mental health concerns (e.g., mood disorders, burnout, and moral injury). They encompassed preventative measures (e.g., self-care strategies; trainings and preparedness; risk-based psychological assessments); post-critical incident supports; direct ongoing mental health care (e.g., counselling delivered in-house, through an employee assistance program (EAP), or with an external provider); and wraparound supports (e.g., fitness programs, nutrition, spiritual care, wellness, mindfulness). Strategies were diverse in intensity and type. For example, in a survey of 177 police detachments in the USA, Ramchand et al. (2018) found four levels of service intensity: (1) *minimal* (e.g., a municipal EAP); (2) *basic* (e.g., some mental health services, critical incident response, training); (3) *proactive* (e.g., in-house mental health care, chaplains, substance use programs, peer support, screening, health and wellness programs); and (4) *integrated* (integration of these services with day-to-day operations). Most of the records endorsed comprehensive services, including preventative and wraparound supports. Developing a suite of services to prevent, treat, and manage occupational stress injuries (e.g., trainings, EAP, peer support, crisis intervention, physical fitness and nutrition programs, chaplaincy, psychological services, and family care) was viewed as important and effective (e.g., Coopie et al., 2019; International Association of Chiefs of Police, 2018a; Sewell, 2021a, c).

Psychological interventions with supporting research evidence were as follows: internet-based cognitive behavioral therapy (iCBT) (Hadjistavropoulos et al., 2021; McCall et al., 2021a, b; Public Safety Canada, 2019); eye movement desensitization and reprocessing therapy (EMDR) (Rodriguez et al., 2023; Sewell, 2021b); and mindfulness-based cognitive interventions (e.g., Clements et al., 2021; Denk-Florea et al., 2020; Murray, 2020; Papazoglou et al., 2021; Rodriguez et al., 2023). One study found mindfulness ineffective for occupational stress among first responders in the UK, however (Wild et al., 2020a, b). Motivational interviewing and problem-solving therapy were also mentioned in the records.

Some commonly recommended services for psychological health and safety, in particular peer support programs and critical incident stress debriefing (CISD), had a weak or lacking evidence base (Ramchand et al., 2018). In theory, peer support reduces stigma and breaks down barriers to support and should be standardized (e.g., Carleton, 2021; Sewell, 2021a); however, the evidence was inconclusive and precise elements of a beneficial peer support program (e.g., formal versus informal; voluntary versus selective) were unclear.

A lack of evidence in support of CISD received discussion in the context of treating post-traumatic stress injuries among police. Despite CISD’s widespread use with public safety personnel, there was little evidence to support it. CISD was recommended in seven of the 98 police-related records (7%) and is therefore included in Table 2. However, three records recommended against its use, citing a lack of evidence (Anderson et al., 2020; Papazoglou et al., 2021; Wild et al., 2020a, b). Papazoglou et al. (2021) noted that the American Psychological Association categorizes psychological debriefing as potentially harmful.

**Table 2:** Summary of recommendations pertaining to police and law enforcement personnel, mapped to the SEM

Recommendation	No. of records included in review
**Individual**	
Promote ***physical health and wellness*** practices; offer in-house access to nutrition and exercise programs, workout facilities, etc.	8
Address ***substance use***	2
**Interpersonal**	
Offer, improve, and standardize ***peer support*** programs	28
Provide access to ***religious, spiritual or chaplaincy*** care	9
Increase ***police-community relationships*** and conduct public outreach programs to foster community trust	8
Adopt ***family-inclusive policies and programs***; work closely with families following the death of an officer	7
**Institutional**	
Offer ***mental health training programs*** for personnel and managers on topics such as preparation for exposure to traumatic material, resilience, mindfulness, suicide prevention and awareness, self-care, and occupational stress	63
Put in place clear ***suicide prevention***, training, and response protocols: e.g., develop an organization-wide suicide awareness and intervention plan; provide suicide prevention, awareness, and intervention training for personnel and leadership; limit access to means of suicide/lethal means of self-harm	26
*** De-stigmatize*** mental health and help-seeking	25
Prevent, address, and reduce ***post-traumatic stress injuries***: screen for post-traumatic stress disorder (PTSD); provide pre-traumatic trainings and preparation; offer peri-traumatic services such as critical incident debriefing and support for acute stress; offer appropriate evidence-based psychological services for PTSD	23
Ensure visibility, presence and care of ***leadership***	18
Increase ***access*** to timely psychological/mental health services and supports (e.g., crisis support; short and long-term counselling; in-house/external psychological services and supports)	18
Ensure ***confidentiality*** of services and supports provided	16
Adopt formal, organization-wide policies, programs and services that ***support equity-deserving populations*** in the workforce (e.g., ***women***, ***2SLGBTQIA+***, ***Northern and Indigenous*** police)	16
Offer targeted, ***evidence-based psychological***, **interventions** (both preventative, and to address occupational stress, post-traumatic stress injuries, and other workplace mental health concerns): e.g., iCBT, mindfulness-based interventions, EMDR	15
Provide ***mandatory psychological interventions*** (e.g., annual or biannual psychological assessments; counselling and/or debriefing)	8
Improve ***manager-to-employee communication***	7
Implement ***critical incident debriefing*** programs* *Note: Three records critiqued critical incident debriefing and recommended to decommission its use due to lack of evidence.	7
Provide/expand access to ***EAP*** programs	6
Support ***pre-retirement and retired police officers*** (ages 60+) through EAP, peer support, benefits, etc.	6
Offer ***return-to-work supports*** following occupational stress injuries	3
Offer ***anti-bullying and anti-harassment*** training programs (including anti-sexual harassment); create anti-harassment policies	2
Offer diversity, equity and inclusion ***training programs*** in the workplace	2
Promote transparent, fair ***scheduling and workload*** in the workplace	1
**Policy**	
Ensure ***presumptive coverage*** for occupational and traumatic stress through policy and legislative levers	4
Expand crisis lines to include professionals with a law enforcement background; consider a ***national crisis line*** for police and law enforcement	1
Promote a ***national public service campaign*** around law enforcement mental health and wellness	1
Improve legislative ***privacy protection*** for officers’ help-seeking	1
Establish a ***hub for research excellence and knowledge translation*** to provide national leadership the mental health of public safety personnel, including police; invest in effective knowledge mobilization	1
Form a ***federal advisory council*** on occupational stress injuries for public safety personnel, including police	1
Adopt a ***national mental health strategy*** for police officers and other public safety personnel	1
Provide financial and community support to ***First Nations and Métis People*** for building a psychologically well workforce; recognize the unique needs of Indigenous and Northern policing communities	1

Reducing workplace stigma was recommended by five records (e.g., Arter et al., 2018; Cohen et al., 2019; Crowe et al., 2022; Drew et al., 2021; Newell et al., 2022). Destigmatizing mental health and help-seeking in the workplace was the fourth most common recommendation for police. Stigma was linked to a culture of not admitting to, and seeking help for, mental health concerns (e.g., Newell et al., 2022). Relatedly, confidentiality was seen as important. Provision of mandatory psychological services was positioned as a means of reducing stigma, given that all officers in an organization would be required to access the same services (e.g., Arter et al., 2018; Taylor, 2022). However, mandatory assessments and/or interventions targeting those with pre-existing risk factors was a concern (Ramchand et al., 2018). Anti-bullying and anti-harassment training, combined with strong workplace anti-harassment policies, were recommended to foster a culture of respect and prevent stigma and bias (e.g., Ricciardelli et al., 2020b).

Other barriers to help-seeking included police officers’ lack of confidence in the relevance and effectiveness of services; fear of being declared unfit for duty; lack of trust in professionals outside policing (i.e., psychologists, counsellors); and perceived or actual professional consequences of accessing services (e.g., demotions) (Ramchand, 2018). Again, enhancing cultures of openness and respect was recommended to improve relational workplace factors (e.g., Edwards et al., 2021; Heber et al., 2020; McCarty et al., 2019; Pitel et al., 2021; Ricciardelli et al., 2020b; Sewell, 2021b).

Workplace protections at an institutional and policy level were also addressed. Some literature called for greater structural supports in response to occupational stressors unique to public safety personnel, such as eroded community-police trust, demands to defund police, and increased workload during the COVID-19 pandemic (Heber et al., 2020). Overall, these recommendations encompassed shifts toward an open and respectful workplace culture (e.g., reduced stigma and bias, visibility and leadership of management related to mental health and wellness, better employee-manager communication), and policy and legal protections (e.g., support for aging and retired officers, fair scheduling and workload, presumptive insurance coverage for workplace psychological injuries, privacy protections) (e.g., Clements et al., 2021; Cohen et al., 2019; Newell et al., 2022).

Specific equity-deserving groups mentioned in the records included women, 2SLGBTQIA+, and Northern and Indigenous police officers (Bastarache, 2021; First Peoples Wellness Circle, 2019; Giwa et al., 2022; Taylor et al., 2022). Sexual harassment and gender-based discrimination were highlighted as problems, including within the RCMP (Bastarache, 2021). Recruitment of equity-deserving populations, inclusive policies, and provision of culturally relevant, equity-focused trainings and education were recommended (Giwa et al., 2022; Mennicke et al., 2018; Taylor et al., 2022). Anti-harassment policies, training, and education initiatives were also mentioned (Bastarache, 2021; Ricciardelli, 2020b; Taylor et al., 2022).

#### Correctional employees

Approximately one-third (*n* = 44, 29%) of records discussed strategies, frameworks, and guidelines in support of correctional employees’ psychological health and safety, including 38 (86%) academic and six (14%) gray records. This occupational grouping included correctional officers and correctional administrators. Correctional officers were discussed in all 44 of the records (100%), correctional administrators were discussed in seven records (16%), and in 20 records (*n* = 44, 45%) correctional personnel were discussed in tandem with other occupations (e.g., police, nurses, lawyers, and probation and parole officers). As with police, mental health trainings, programs, services, and wraparound supports were most often recommended (see Table 3).

**Table 3:** Summary of recommendations pertaining to correctional personnel, mapped to the SEM

Recommendation	No. of Records Included in Review
**Individual**	
Develop and promote ***healthy lifestyle habits and coping*** among staff, e.g., cognitive strategies (reframing, acceptance, problem-solving), alternatives to substance/alcohol use, exercise and physical activity	8
**Interpersonal**	
Offer ***mentorship and/or clinical supervision opportunities***, i.e., spaces to discuss emotions, workplace incidents, learnings and insights	10
Encourage ***positive interactions between correctional officers and offenders***; improve prisoner quality of life to support positive relationships and avoid escalating tensions	8
Provide ***peer support***, e.g., lunchtime drop-in sessions; consider standardizing and/or formalizing peer support programs	7
*** Support, recognize, and celebrate staff***, e.g., host on-site celebrations and opportunities to socialize, hold employee recognition events, etc.	6
Provide a designated ***religious and spiritual care*** provider	1
Improve ***communication and connectedness with the surrounding community***	1
**Institutional**	
Offer ***workplace mental health training*** on topics such as resilience, preparedness, and mental health first aid; consider adopting standardized, evaluated training programs, e.g., AMStrength, Road to Mental Readiness (R2MR), Before Occupational Stress	30
Increase access to mental health resources, services, programs, and interventions by adopting ***dedicated on-site mental healthcare services for employees*** (e.g., appoint a designated on-site psychologist)	14
Prevent, address, and reduce ***post-traumatic stress injuries***: provide training on workplace exposure to traumatic stress; offer and standardize crisis and trauma support interventions, e.g., trauma-informed psychological services, critical incident stress management programs	13
Improve ***manager-to-employee communication***	10
Improve hiring practices, e.g., explore ***resilience screening*** at time of hiring/enrollment (and address associated ethical and privacy concerns)	9
Offer ***evidence-based psychological interventions*** to prevent and address post-traumatic stress injuries and other mental health concerns, including virtual/e-therapies (e.g., iCBT)	5
Offer designed ***employee breakrooms*** separate from the working space	5
*** Destigmatize*** mental health and help-seeking	5
Ensure visibility and presence of ***leadership***	5
Address ***understaffing*** in the workplace: ensure proper budget allocation for hiring, staffing, backfilling roles when staff are on leave, etc.	5
Audit and improve ***physical environment of prisons*** (i.e., overcrowding, poor sanitation, dim lighting, lack of fresh air)	4
Offer ***anti-bullying and anti-harassment*** training programs; create strong anti-harassment policies; adopt a safe and formal way to report grievances	4
Adopt ***flexible work schedules*** to encourage work-life balance; ensure scheduling is transparent and fair	2
Provide and expand access to trauma-informed ***EAP*** services	2
Implement confidential, evidence-based ***psychological assessments*** of staff mental health	2
Facilitate ***return-to-work*** programs following occupational stress injuries	2
Address ***hyper-masculinity*** in the workplace; develop tools and strategies to encourage men to be proactive in maintaining a respectful work culture	2
Provide workplace supports for members of the ***2SLGBTQIA*** + community	1
Offer ***equity and diversity training***	1
Appoint an ***equity and diversity officer***	1
Increase ***pay and benefits***	1
**Policy**	
Ensure ***presumptive coverage*** for occupational and traumatic stress through policy and legislative levers	2
Establish a ***hub for research excellence and knowledge translation*** to provide national leadership on the mental health of public safety personnel, including correctional officers; invest in effective knowledge mobilization	1
Form a ***federal advisory council*** on occupational stress injuries for public safety personnel, including correctional officers	1
Adopt a ***national mental health strategy*** for correctional officers and other public safety personnel	1
Address the needs of ***small, rural, isolated, and/or First Nations communities***	1

At an individual level, self-care, including healthy coping measures for stress (e.g., cognitive strategies, exercise, nutrition), was seen as important to protect against occupational stress and mental health concerns (e.g., Jaegers et al., 2020; Johnston et al., 2022). Other institutional-level recommendations concentrated on the importance of psychological preparedness for working in prisons, jails, detention centers, and other correctional facilities. These recommendations pertained to services and supports to prevent and address occupational stress, burnout, compassion fatigue, psychological injury (including post-traumatic stress injuries), and other mental health concerns among correctional personnel. Records mentioning mental health trainings often discussed the benefits of evidence-based resilience and preparedness programs such as AMStrength, Road to Mental Readiness (R2MR), and Before Occupational Stress (Johnston et al., 2023; Ricciardelli et al., 2021a; Siqueira Cassiano, 2022; Stelnicki et al., 2021).

As with police, a suite of comprehensive services encompassing peer support, EAP, and counselling was viewed as beneficial. Internet-based cognitive behavioral therapy (iCBT) was found to have beneficial outcomes (e.g., McHall 2021a; McHall, 2021b). Providing dedicated on-site mental health care for staff, such as an on-call staff psychologist, was a recommendation for correctional personnel not identified as often in the police literature. Unlike police, who work in the community, correctional officers remain in the same facility when working. Embedding services on site was seen as a means of increasing access, including directly after a critical incident (e.g., Eades, 2020; Dennard et al., 2021; Siqueira Cassiano et al., 2022; Ricciardelli, 2023a).

Some recommendations related to relationship-building and compassionate leadership. The need to improve manager-employee communication was a common recommendation. Encouraging positive relationships among correctional officers and people in their care was also noted; some literature linked de-escalation among staff and justice-involved individuals as a mutually beneficial outcome i.e., improving prisoner quality of life also improved employees’ experiences at work (Ricciardelli et al., 2023a).

Greater opportunities for self-reflection, including mentorship and clinical supervision, were noted as promising (e.g., Dennard et al., 2021). Supervision and mentorship were recommended as opportunities to share emotions, process workplace incidents, discuss ethical matters, and share learnings and insights. In theory, this could benefit correctional officers and become a means of encouraging trauma-informed practices in the workplace.

The practice of conducting psychological assessments during recruitment, hiring, or enrollment to assess for risk factors such as suicidal ideation, substance use, etc., was recommended in one record (Ferdik & Pica, 2023), but without clear evidence. On the one hand, screening was viewed as positive for the organization from a risk-based perspective. On the other hand, privacy and ethical implications could emerge. Confidential, third-party/external, evidence-based resilience screenings were seen as essential. Further consideration and exploration with this practice was recommended (Public Safety Canada, 2019).

Other institutional-level recommendations included improving facilities (e.g., addressing sanitation, providing access to technology, offering dedicated employee breakrooms separate from the work space); increasing hiring/staffing to address under-resourcing; increasing pay and benefits; adopting flexible and fair work schedules for staff; ensuring fair allocation of work; and reducing burdensome administrative labor (e.g., Dennard et al., 2021; Ferik & Pica, 2023; Ricciardelli et al., 2018a, 2020a, 2023a; Siqueira Cassiano et al., 2022). Leadership and management were also called upon to be visible in support of mental health and wellness, communicate clearly and fairly, and recognize staff members (e.g., by hosting events and celebrations) (e.g., Dennard et al., 2021).

At the policy level, similar to police and law enforcement, presumptive coverage was mentioned as a lever to support correctional employees experiencing traumatic stress. In Canada, presumptive coverage policies have been successfully implemented across some provinces, including Manitoba, Ontario, and British Columbia. Other jurisdictions, such as New Brunswick, remain without legislation that recognizes traumatic stress as a work-related injury among correctional employees (National Union of Public and General Employees, 2024 ; WorkSafeNB, 2023).

Equity-deserving groups addressed in the records included women, 2SLGBTQIA+, and First Nations (Burdett et al., 2018; Mennicke et al., 2018; Sapers et al., 2018). One record noted that correctional staff identifying as women are more likely to seek support for mental health challenges and demonstrate less stigma towards others living with mental illness than their male colleagues (Ricciardelli et al., 2021b). A correctional workplace culture of hyper-masculinity was identified in one study regarding the experiences of women correctional officers (Burdett et al., 2018). To support 2SLGBTQIA + officers, Mennicke et al. (2018) recommended inclusive policies and procedures, support groups, and 2SLGBTQIA+-affirming community events. Equity and diversity training for employees and management was also suggested.

#### Legal employees and those directly involved in court proceedings

Within the 150 records, 16 (11%) discussed strategies, frameworks, and guidelines for supporting the psychological health and safety of legal employees and those whose roles brought them in direct contact with court proceedings (e.g., jurors), mostly from the gray literature (12, 75%), with four (25%) from the academic literature. These records represented: lawyers (12 records), judges (four records), paralegals/law clerks and assistants (four records), mental health court workers/court diversion workers (one record), and jurors (one record). Gladue writers were included in the searches performed but no related records were found. Nearly all records (15, 94%) focused on legal occupations, while one (6%) grouped legal and court-related roles with other public safety personnel (e.g., correctional and police officers).

Most strategies, frameworks, and guidelines in these records sought to address and improve mental health and substance use outcomes (e.g., burnout, compassion fatigue, mood disorders, occupational stress, addictions, and suicidal ideation). Strategies were framed in terms of wellness as a professional competency (e.g., Task Force on Wisconsin Lawyer Well-Being, 2021). Institutional-level trainings (also described as education, workshops, seminars, or professional development) were the most recommended strategy, as seen in Table 4. Topics mentioned in the records included help-seeking, resilience, stress, stressors (e.g., anxiety, money, relationships), and the mental health of law students and jurors. Other resources mentioned were mentorship, peer support, and mindfulness; online/virtual access to counselling and other services was also viewed as helpful. Two records mentioned de-emphasizing alcohol use at professional events, linked to an entrenched culture of drinking and permissiveness around alcohol as a legal drug (National Task Force on Lawyer Well-Being, 2017; Supreme Judicial Court Standing Committee on Lawyer Well-Being, 2021). Several records mentioned the need for education and awareness about substance use. Organizations such as bar associations were called upon to provide access to treatments and resources.

**Table 4:** Summary of recommendations in support of legal employees, mapped to the SEM

Recommendation	No. of Records Included in Review
**Individual**	
* Promote* ***healthy lifestyle habits***	1
**Interpersonal**	
Provide greater ***mentorship*** opportunities for legal professionals	6
Adopt a ***peer support*** network for lawyers and judges	2
De-emphasize ***alcohol use at social events and parties***	2
**Institutional**	
Develop and offer ***trainings, seminars, workshops, and professional development opportunities*** for legal professionals and students on mental health-related topics, e.g., resilience, substance use/addictions, anxiety, relationships, money management, nutrition, wellness, available resources, when to seek help	15
Incorporate ***wellness*** in all aspects of the legal profession, e.g., implement proactive wellness initiatives within legal organizations; create and oversee wellness action plans; conduct wellness surveys	13
Provide resources, supports, and services related to ***alcohol/substance use and addiction***	7
*** De-stigmatize*** mental health and help-seeking within the legal profession	5
Demonstrate strong ***organizational/leadership commitment*** to mental health	4
Fund and support ***lawyer assistance programs*** and insurance coverage for psychological services	4
Initiate conversations about ***suicide awareness and intervention*** in the workplace; consider a suicide intervention program/plan	4
Provide ***online/virtual*** access to wellness initiatives, counselling, peer support, etc.	3
Provide ***education and training*** on equity and diversity, cultural competency, etc.	3
Ensure ***confidentiality*** of services and supports provided	2
Create ***anti-bullying*** policies and initiatives	2
Provide ***mindfulness*** tools, education, and resources	2
Provide ***post-jury duty support***, debriefing, and confidential counselling; provide information to jurors about the potential psychological impacts of jury duty	1
Recognize and support ***sex crime prosecutors’ efforts to support survivors***	1
Respect employees’ ***work-life boundaries***	1
Develop programs and services to support those in ***rural, remote, and isolated communities***	1
Create ***gender-inclusive workplaces***, e.g.: hold a zero-tolerance policy for gendered bullying, include women as leaders, offer flexible schedules to attract women employees	1

Awareness, education, and de-stigmatization of help-seeking were discussed as important measures for legal employers, organizations, and professionals (e.g., National Task Force on Lawyer Well-Being, 2017). As with police, concerns about stigma and the potential consequences of taking a leave, seeking services, or admitting the need for help were commonly raised. Furthermore, provision of benefits, insurance, and access to lawyer assistance programs were seen as essential (National Task Force on Lawyer Well-Being, 2017).

One academic record discussed women paralegals, clerks, and legal assistants, and the issue of gender equality in legal workplaces (Yu, 2017). Yu recommended promoting women as leaders/managers and offering flexible working hours to attract women employees. The need for education and training on equity and diversity was also mentioned in a few records. No records in this occupational grouping made recommendations related to policy, legislation, or broader structural reforms.

#### Correctional and forensic mental health and health care providers

Of the 150 records, 10 (7%) discussed strategies, frameworks, and guidelines for supporting the psychological health and safety of mental health and health care providers, including nine (90%) academic and one (10%) gray. Occupations represented were forensic and correctional nurses (10 records), forensic and correctional psychologists (four records), forensic and correctional psychiatrists (three records), social workers in forensic settings (two records), and psychometrists (two records). The literature addressed the workplace settings of forensic and psychiatric hospitals; recommendations were primarily for hospital leaders and administrators, with the bulk of strategies pertaining to psychiatric and forensic nurses. Six (60%) focused on mental health and health care professions alone. The other four (40%) discussed these roles along with other occupations involved in corrections and justice work (e.g., correctional officers, probation and parole officers, police officers).

The most common institutional-level recommendations for supporting the psychological health and safety of nurses and other health care providers related to provision of workplace mental health training, along with psychological services, supports, and interventions (see Table 5). One study (Hilton et al., 2020) noted the importance of evidence-based, responsive, available psychological interventions following potentially traumatizing incidents, along with access to mental health supports such as counselling. Another key recommendation was to designate a mental health professional and peers to support employee mental health (Bouchard, 2022; Dennard et al., 2021; Hilton et al., 2020, 2022; Rodrigues et al., 2021a, b).

**Table 5:** Summary of recommendations in support of correctional and forensic employees, mapped to the SEM

Recommendation	No. of Records Included in Review
**Individual**	
* Promote* ***healthy living initiatives*** *to deal with stress*	1
**Interpersonal**	
Develop ***peer support*** programs for providers working in forensic and correctional mental health care (e.g., nurses, psychologists, psychiatrists, social workers)	4
**Institutional**	
Provide ***workplace mental health training*** on topics to support employee psychological health and safety, e.g., stress reduction and management, psychological first aid	5
*** Designate a mental health professional*** to serve employee mental health within psychiatric hospitals and forensic settings	4
*** Support staff to prevent and safely respond to workplace critical events*** (e.g., screen every patient for suicide risk, inpatient violence risk, and risk of seclusion; increase breadth of nonviolent crisis intervention training with an emphasis on de-escalation)	3
Provide a ***psychologically healthy workplace*** with an emphasis on workplace trauma prevention; offer wellness initiatives and increased mental health support	3
Facilitate strong ***return-to-work programs*** following occupational stress injuries	2
Ensure nurses and allied health professionals receive ***detailed, onsite professional training regarding mental disorders and their treatment***, including relevant psychotherapeutic and behavioral interventions	1
*** Designate an occupational health and safety professional*** to be the lead for workplace trauma within psychiatric hospitals	1
*** Destigmatize*** mental health and help-seeking among health care workers	1
Provide employees with ***clinical supervision and/or other opportunities for self-reflective, trauma-informed practice*** (e.g., drop-in sessions, peer-vision)	1
Improve ***employee-to-manager communication***	1
Provide opportunities for employees to engage in ***areas of work they find rewarding***	1
Offer competitive ***pay and benefits***	1
Afford employees greater control over their ***workload***	1
Implement ***evidence-based critical event operational debriefing*** following a critical incident in the workplace	1
Offer ***evidence-based mental health services and interventions*** to any employee who witnesses, responds to, or is exposed to the details of a critical incident	1
**Policy**	
Work with unions and the ***Workplace Safety and Insurance Board (WSIB)*** to create a user-friendly, transparent, consistent process for WSIB claims following an occupational stress injury	1

Institutional and workplace policies within hospitals were highlighted as necessary for ensuring safety and equipping staff to respond to critical incidents. For example, policies requiring forensic patients to be screened for risk of violence and suicidal ideation were mentioned multiple times. Other recommendations concerned supports for employees who suffered occupational or post-traumatic stress injuries following a workplace critical incident. Institutional supports for employees with post-traumatic stress or other occupational stress injuries included designating an onsite professional to be the occupational health and safety lead for workplace trauma within psychiatric hospitals; facilitating strong return-to-work programs; and simplifying the process for worker insurance claims following a psychological injury (Hilton et al., 2020).

For example, at the policy level, Hilton et al. (2020) address the challenges psychiatric hospital staff face when dealing with workplace trauma and the complexities of navigating the workplace insurance claims process. They recommend that psychiatric hospitals collaborate with unions and their respective workplace insurance boards to create user-friendly, transparent, and consistent processes for claims, thereby reducing administrative burdens on employees. Implementing such policies can enhance trust between injured workers and employers and support more effective workplace injury management systems (Hilton et al., 2020).

No records in this occupational category explicitly discussed issues related to equity and diversity, nor priority populations for the scoping review (e.g., women, 2SLGBTQIA+, BIPOC).

#### Criminal justice system support workers

Within the 150 records, eight (5%) discussed strategies, frameworks, and guidelines for supporting the psychological health and safety of those captured in this occupational grouping, including probation and parole officers (six records), victim advocates (one record), and mobile crisis response staff (one record). Of these records, there was a blend of academic and gray literature: three academic studies (38%) and five gray literature reports (62%).

The records focused on settings in which probation and parole officers conduct their duties (e.g., courts, correctional facilities, young offender correctional facilities). Thus, some records overlapped with those within the correctional personnel grouping discussed above. In all, only two records (25%) pertained to probation and parole officers alone. Four records (50%) grouped probation, parole, and mobile crisis workers with other occupations (e.g., correctional officers, police officers and law enforcement, forensic and correctional nurses, lawyers, paralegals).

Interpersonal and institutional strategies for these populations included providing workplace mental health trainings for probation and parole officers, along with team-building opportunities and direct access to psychological services. As with other occupational groupings, education, awareness, and access to services were deemed important (see Table 6).

**Table 6:** Summary of recommendations in support of criminal justice system support workers

Recommendation	No. of Records Included in Review
**Individual**	
* Promote* ***healthy living initiatives*** *to deal with stress*	1
**Interpersonal**	
*** Pair parole officers together*** when visiting high-risk clients	1
Offer ***team-building*** exercises, group workshops, and shared educational opportunities in the workplace for parole and probation officers	1
**Institutional**	
Provide ***workplace mental health training*** (e.g., on resilience) for parole and probation officers and others whose work liaises with the criminal justice system	6
Ensure ***full workload coverage*** for parole officers on leave	2
*** Destigmatize*** help-seeking, mental health, and taking leave when needed	2
Provide ***direct access to psychological services*** for employees (e.g., counselling, psychological assessment and intervention)	2
Designate an ***on-site wellness space*** for employee physical and psychological wellbeing	1
Ensure victim advocate organizations are ***mindful of client-facing hours***	1
*** Increase the number of parole officers*** working in both the community and institutions	1
Grant ***paid overtime*** to community parole officers, who regularly work extra hours	1
**Policy**	
Form a ***federal advisory council*** on occupational stress injuries for public safety personnel, including parole and probation officers	1
Explore protective legislative measures, including ***presumptive coverage*** for occupational stress injuries	1
Include parole and program officers and dispatch officers in the term “***public safety officers***”	1

Records emphasized the heavy workloads of probation and parole officers, who often work unpaid overtime and lack appropriate replacement coverage when on leave. Occupational stress was linked to high volumes of work, unreasonable deadlines, and low staffing (e.g., Ricciardelli et al., 2022b; Union of Safety and Justice Employees, 2019). A report from the Union of Safety and Justice Employees (2019) called upon Correctional Service Canada to increase the number of parole officers working in the community due to chronic understaffing and an increase in officers’ work duties. The recommended changes included appropriate coverage for officers who go on leave, greater staffing allocations, and granting of paid overtime. Other recommendations related to improving workplace culture, e.g., by offering an on-site wellness space or opportunity for education. One study noted that the probation and parole workplace culture was perceived as reactive, hierarchical, and unsupportive (Ricciardelli et al., 2022b).

At the policy level, some records discussed tensions around the inclusion of probation and parole officers in the more widely recognized category of public safety personnel (PSP) (e.g., police and law enforcement; correctional officers). A report to the House of Commons on occupational stress injuries among PSP, for example, called for the inclusion of parole and program officers in the term “public safety officer” (Oliphant, 2016). The Union of Solicitor General Employees’ report *Moving Forward* (2017) grouped probation and parole officers within the broader category of federal public safety workers. The inclusion of probation and parole officers in the broader PSP category would allow for more comprehensive health leave protections as part of presumptive coverage legislation for psychological injuries sustained in the workplace (National Union of Public and General Employees (2024) . As of June 2024, the Workers Compensation Act in British Columbia, Canada was amended to include presumptive coverage for probation and parole officers, but other jurisdictions still lack similar protections (WorkSafeBC, 2024).

No records in this occupational category discussed equity and diversity, nor priority populations for the scoping review (e.g., women, 2SLGBTQIA+, BIPOC).

## Discussion

This review synthesized common recommendations from the literature and mapped them to the SEM as a way of contextualizing the wide range of services and supports that can protect the psychological health and safety of people who work in the criminal justice system. Organizing sources of accountability through the SEM supported the benefits of frameworks, strategies, interventions, and guidelines found in this review, ensuring a multi-level, comprehensive, system-wide approach to future implementation.

The findings of this review demonstrate the importance of a shared responsibility among multiple actors and levels for safeguarding the psychological well-being of criminal justice workers. Individuals within the system are encouraged to prioritize self-care, show respect, and offer support to colleagues. Mentors, peers, and supervisors play a vital role by exemplifying healthy communication, providing interpersonal assistance, combating stigma, and advocating for systemic improvements. Institutional leaders, including employers, unions, and governmental bodies, possess the authority to implement effective strategies and best practices. Furthermore, policymakers, researchers, and system stakeholders contribute to the process of change by offering expert guidance and other forms of support.

Although the benefits of collective responsibility have traditionally focused on patient populations, the current review extends its relevance in criminal justice workers (Hean et al., 2017). Findings from this review and prior research suggest that transformational leadership styles and interactions among criminal justice workers can influence employee mental health (Cho, 2023). Indeed, in both criminal justice workers and other professions, social support is one of the strongest predictors of mental health outcomes and burnout (Zeng et al., 2020).

By far, the most cited recommendation across all populations and occupational groupings was the provision of mental health-related trainings in the workplace. Training and education were seen as a means of breaking down stigma, thereby encouraging help-seeking; preparing personnel for exposures to potentially traumatic incidents and material; reducing workplace harassment; and promoting trauma-informed and culturally responsive approaches when working with justice-involved individuals, forensic and other patients, victims/survivors, family members, individuals living with mental illness, and other community members.

The second most recommended strategy was the provision of psychological interventions for preventative and acute/crisis purposes. While the importance of these types of interventions is frequently highlighted among criminal justice workers (Fusco et al., 2021), the current review centered on specific supports and services that may benefit workers as part of broader organizational strategies. Services include counselling, psychological assessment (e.g., regarding suicidal ideation, underlying risk factors, etc.), critical incident debriefing, and peer support. Peer support emerged as a recurring theme across all occupational groups, with variations tailored to specific contexts. For example, law enforcement emphasized external, confidential peer networks, whereas corrections recommended on-site, trauma-informed peer programs. Despite these differences, the overarching goals of fostering resilience and reducing stigma were consistent across all occupational categories.

Moreover, the types of beneficial services and supports may differ by criminal justice profession. For instance, a key recommendation within the correctional literature was providing dedicated on-site psychological services (e.g., a staff psychologist). Conversely, with police and law enforcement, access to external providers was sometimes seen as advantageous due to increased privacy and confidentiality. With lawyers, online/virtual services were often recommended. These differences exist across populations owing to the particularities of workplace settings, work duties and responsibilities, and workers’ individual needs. Independent of interventions, the need for wraparound care encompassing prevention, promotion, early intervention, peer and family support, physical wellness, and spiritual care was often highlighted in the findings across all occupational groupings.

Strategies, guidelines, and frameworks beyond an individual level are an integral aspect of the wraparound approach. Unions and researchers advocated for workers to have fair workloads and schedules, access to leave when needed, physically safe working conditions, and workplaces free of discrimination and harassment. At the policy and systems levels, presumptive coverage was highlighted as a lever to support workers exposed to occupational stressors and traumatic incidents. Policies to establish adequate funding—e.g., to increase staffing levels among correctional and probation and parole personnel—were also discussed. Most records captured in this review were concentrated on strategies to be undertaken by individual employees or their employers, i.e., not at the provincial/state or federal/national levels.

Overall, across all occupational groupings, there was a limited scope of recommendations at these broader policy and system levels. For example, only two reports highlighted federal strategies for Canada to address occupational stress among public safety personnel, with recommendations involving establishing a hub of research excellence, engaging in knowledge translation, and forming a federal advisory council (Public Safety Canada, 2019; Oliphant, 2016). Insufficient attention to the policies and systems enabling implementation of institutional recommendations may contribute, at least in part, to the ongoing mental health challenges and low perceived support observed in criminal justice workers (Moghimi et al., 2022b).

The review identified a few noteworthy barriers that contribute to poor or inconsistent implementation of mental health strategies in correctional environments. Lack of consistent funding was noted as a recurring issue that hinders the establishment and sustainability of programs, services, and supports (Canada et al., 2021; National Officer Safety Initiatives, 2018; Oliphant, 2016). Organizational resistance was named as another challenge, particularly in settings where mental health support is not a cultural norm. For instance, Ricciardelli et al. (2023a) and Ricciardelli et al. (2022b) highlight that correctional environments often operate under rigid structures, making it difficult to integrate onsite psychological services or training programs. Additionally, stigma and discrimination remain pervasive obstacles, as correctional officers and police, for example, often associate seeking mental health services with weakness (Miller, 2022; Ricciardelli et al., 2018c; Rodriguez et al., 2023). Jurisdictional fragmentation also complicates implementation, leading to inconsistencies in program delivery or policy implementation (Public Safety Canada, 2019; Ricciardelli et al., 2021a).

Several strategies were proposed to address these barriers. To mitigate funding constraints, Rodrigues et al. (2021b) and Fortune et al. (2018) recommend scalable, cost-effective approaches like peer support programs and networks, which can utilize existing personnel to foster collective support and reduce stigma. To address organizational resistance, Ricciardelli et al. (2021a) and Violanti (2017) emphasize the need for leadership engagement and training for supervisors to champion mental health programs. Additionally, stigma-reduction strategies can normalize seeking help and highlight the benefits of mental health support (Drew & Martin, 2021; Ricciardelli et al., 2021b). Developing standardized guidelines across jurisdictions can address fragmentation and support the consistent implementation of evidence-based practices (Carleton, 2021; Sewell, 2021a). Finally, advocacy for sustained funding, supported by outcome evaluations that demonstrate program efficacy, is critical to achieving long-term, systemic change across all criminal justice settings and professions (Burgess, 2018; Housefather, 2018; Oliphant, 2016; Stelnicki et al., 2021).

### Limitations

Although the review has several strengths, it is important to acknowledge and address its limitations. First, apart from a few studies validating psychosocial interventions (e.g., iCBT, mindfulness, resilience training), the strategies, frameworks, and guidelines mapped in this report often lacked substantial long-term evaluative data and/or iterative development of the recommended approach over time. For example, the evidence on the benefits of peer support and critical incident debriefing was limited and sometimes contradictory, and in some instances found these practices to be harmful (e.g., Anderson et al., 2020; Wild et al., 2020a, b) or their implementation poor (e.g., Beshai et al., 2016), despite being widely recommended across the continuum of supports for criminal justice workers (e.g., Ferdik & Pica, 2023; Rodrigues et al., 2021b). The review provides a summary of current recommended approaches, but based on our scoping review methodology, we were unable to weigh the evidence for each approach. Further studies are needed to evaluate implementations of recommended approaches, and future reviews should conduct meta-analyses of their effectiveness.

Second, this review captured a significant number of gray literature and non-peer-reviewed documents. While academic literature focuses on empirical and theory-driven research, gray literature often addresses broader, applied topics, making it valuable for identifying common principles, ongoing trends, and policy frameworks. However, the variability in quality and lack of standardized evaluation approaches in gray literature underscore the need for careful appraisal in future reviews.

Third, the concentration of data on police and law enforcement and correctional personnel may have biased the recommendations and overall focus of the study. As well, there were limited results from Scandinavian countries, despite this being an inclusion criterion for the review. Given that most of the records were from the USA and Canada, the recommendations synthesized from the review may not adequately account for the unique cultural, organizational, and systemic factors influencing mental health support in other regions, potentially limiting the applicability of the findings to a broader international audience. These limitations underscore the importance of conducting more comprehensive and inclusive research that encompass a wider range of occupational groups and geographic regions within the criminal justice system.

Fourth, due to the focus on overall mental health, substance use and related terms were not included in the search strategy. Accordingly, substantial substance use recommendations were not explicitly extracted. Those that appeared in the findings supported lawyers, judges, and paralegals. Future reviews should consider integrating substance use into strategies related to psychological health and safety for these populations and within these occupational contexts.

### Future directions

Given the low number of records addressing equity and diversity, an important future direction is applying this data to marginalized populations. The prevalence of mental health concerns among marginalized populations generally may amplify difficult outcomes for people working in or adjacent to the criminal justice system who face structural forms of oppression (Batton et al., 2019; Martinez, 2019). Narrowing in on strategies to support populations such as Indigenous People, racialized populations, and 2SLGBTQIA + is necessary to expand and refine the findings. While some records discussed gender-based barriers and harassment experienced by women and 2SLGBTQIA+, there is considerable room to expand this type of research.

Future studies could also address implementation and evaluation of the strategies captured in the review. There were a limited amount of evaluative data in the records extracted, particularly regarding policy-level recommendations. Future research could draw upon implementation science and evaluation methodologies to investigate the efficacy of specific strategies at institutional, provincial, and/or federal levels. For example, recommendations related to funding (e.g., for staffing and training) and structural benefits and coverage (e.g., presumptive coverage, employee/lawyer assistance programs, and WSIB) deserve future evaluation to better understand needs, outcomes, and gaps.

Future studies could also aim to better understand the interconnectedness of the mental health of people involved in the criminal justice system and the psychological health and safety of people who work in the criminal justice system. Only a few records captured in the review discussed this relationship. For example, future research could explore how fostering trusting relationships between inmates and correctional officers might be mutually protective against psychological injuries, how improving and protecting the mental health of inmates might create more psychologically safe workplaces, or how improving relationships between police and local communities can improve the mental health of law enforcement personnel.

## Conclusion

Employees who work in the criminal justice system face unique risks, including exposures to violence and compounding occupational stressors. This scoping review identified several intersecting recommendations for interventions and strategies to protect and promote the psychological health and safety of people who work within, or whose work directly relates to, the criminal justice system. The most common strategies included training, provision of evidence-based psychological interventions, support for acute stress, peer support, mentorship, de-stigmatization of mental health and help-seeking, increased connectedness with surrounding communities, adequate funding to maintain safe and supportive workplace environments, and presumptive coverage for occupational stress. The review captured a relative lack of system and policy-level recommendations overall. For the most part, the records included in this review addressed individual and institutional-level services and supports such as internet-based cognitive behavioral therapy (iCBT), mindfulness, resilience trainings, critical incident stress debriefing (CISD), peer support, and compassionate employee-manager communication. Fewer records sought to address and ameliorate the structural factors related to psychological health and safety in the workplace, including understaffing, labor shortages, low wages, patchy mental health benefits, underfunding, racism, discrimination, and sexism affecting all levels of the SEM. Future studies could include prevalence data, qualitative research, and reports on the experiences of racialized, Indigenous, rural/Northern, and other marginalized populations within the occupational sub-groupings captured in this review.

## Supplementary Information

Supplementary Material 1.

## Data Availability

Supplemental Table 4 cites all records included in this scoping review, which are published in peer-reviewed journals or freely available online.

## References

[CR1] Abeyta, S. (2021). An exploratory examination of the effects of workplace strain on correctional officers. *Deviant Behavior*, *44*(1), 75–92. 10.1080/01639625.2021.2012093

[CR2] Anderson, G. S., Di Nota, P. M., Groll, D., & Carleton, R. N. (2020). Peer support and crisis-focused psychological interventions designed to mitigate post-traumatic stress injuries among public safety and frontline healthcare personnel: A systematic review. *International Journal of Environmental Research and Public Health*, *17*(20), 7645. 10.3390/ijerph17207645 33092146 10.3390/ijerph17207645 PMC7589693

[CR3] Arksey, H., & O’Malley, L. (2005). Scoping studies: Towards a methodological framework. *International Journal of Social Research Methodology*, *8*(1), 19–32. 10.1080/1364557032000119616

[CR4] Arter, M. L., & Menard, K. S. (2018). An examination of the reasons police officers fail to seek treatment for occupational stress. *Law Enforcement Executive Forum*, *18*(1), 30–42.

[CR5] Baral, S., Logie, C. H., Grosso, A., Wirtz, A., & Beyrer, C. (2013). Modified social ecological model: A tool to guide the assessment of the risks and risk contexts of HIV epidemics. *Bmc Public Health*, *13*(482), 1–8. 10.1186/1471-2458-13-482 23679953 10.1186/1471-2458-13-482 PMC3674938

[CR6] Bastarache, M. (2021). Broken dreams, broken lives: The devastating effects of sexual harassment on women in the RCMP. *A final report on the implementation of the Merlo Davidson settlement agreement*. https://www.rcmp-grc.gc.ca/wam/media/4773/original/8032a32ad5dd014db5b135ce3753934d.pdf

[CR7] Batton, C., & Wright, E. M. (2019). Patriarchy and the structure of employment in criminal justice: Differences in the experiences of men and women working in the legal profession, corrections, and law enforcement. *Feminist Criminology*, *14*(3), 287–306. 10.1177/1557085118769749

[CR8] Beshai, S., Carleton, R. N., Dirkse, A. D., Duranceau, A., Hampton, D. J. A., Ivens, E. S., LeBouthillier, M. D., Tamaian, A., Sapach, M. J. N., Thorisdottir, S. A., Walker, L. K., & Wuerch, A. M. (2016). *Peer support and crisis-focused psychological intervention programs in Canadian first responders: Blue paper*. Retrieved from https://www.justiceandsafety.ca/rsu_docs/blue_paper_full_web_final_production_aug_16_2016.pdf

[CR9] Bouchard, L., Williams, D., Kiser, L., Freese, E., & Taren, D. (2022). Promoting professional quality of life and resiliency in sexual assault nurse examiners. *Journal of Forensic Nursing*, *18*(1), 13–20. 10.1097/JFN.0000000000000350 35170881 10.1097/JFN.0000000000000350

[CR10] Buchanan, B., & Coyle, C. J. (2017). *National task force on lawyer well-being: Creating a movement to improve well-being in the legal profession *[Report]. American Bar Association, 1–73. https://lawyerwellbeing.net/wp-content/uploads/2017/11/Lawyer-Wellbeing-Report.pdf

[CR11] Burdett, F., Gouliquer, L., & Poulin, C. (2018). Culture of corrections: The experiences of women correctional officers. *Feminist Criminology*, *13*(3), 329–349. 10.1177/1557085118767974

[CR12] Burgess, M. (2018). *Inquiry into the role of commonwealth, state and territory governments in addressing the high rates of mental health conditions experienced by first responders, emergency service workers, and volunteers *[Inquiry report]. Police Federation of Australia. https://pfa.org.au/wp-content/uploads/2021/08/sub80_PFA-First-Responders.pdf

[CR13] Cadieux, N., Cadieux, J., Youssef, N., Gingues, M., & Godbout, S. M. (2020). *Research report: A study of the determinants of mental health in the workplace among Quebec lawyers phase II – 2017–2019 *[Report] (pp. 1–177). Université de Sherbrooke Business School.

[CR14] Canada, K. E., Watson, A. C., & O’kelley, S. (2021). Utilizing crisis intervention teams in prison to improve officer knowledge, stigmatizing attitudes, and perception of response options. *Criminal Justice and Behavior*, *48*(1), 10–31. 10.1177/0093854820942274

[CR15] Carleton, N. (2021). Collaborating to support the mental health of public safety personnel: The Canadian Institute for Public Safety Research and Treatment. *Canadian Psychology*, *62*(2), 167–173. 10.1037/cap0000267

[CR16] Cho, C. C. (2023). A Cross-level Study of the consequences of work stress in police officers: Using transformational Leadership and Group Member interactions as an example. *Psychology Research and Behavior Management*, *16*, 1845–1860. 10.2147/PRBM.S413075 37223307 10.2147/PRBM.S413075 PMC10202197

[CR17] Clements, A. J., Sharples, A., & Kinman, G. (2021). Identifying well-being challenges and solutions in the police service: A world café approach. *The Police Journal*, *94*(2), 81–101. 10.1177/0032258X19898723

[CR18] Cohen, M. I., McCormick, V. A., & Rich, B. (2019). Creating a culture of police officer wellness. *Policing: A Journal of Policy and Practice*, *13*(2), 213–229. 10.1093/police/paz001

[CR19] Coopie, C., Coopie, J., Drake, J., Joyce, N., Robinson, M. J., Smoot, S., Stephens, D., & Villaseñor, R. (2019). *Law enforcement and mental health and wellness programs: Eleven case studies*. Washington, DC: Office of Community Oriented Policing Services. https://portal.cops.usdoj.gov/resourcecenter/RIC/Publications/cops-p371-pub.pdf

[CR20] Crowe, A., Averett, P., Bonner, H., & Franks, C. (2022). Let them know it’s okay to get help: Addressing the mental health needs of police officers. *Administration and Policy in Mental Health and Mental Health Services Research*, *49*(4), 613–622. 10.1007/s10488-022-01187-1 35000040 10.1007/s10488-022-01187-1

[CR21] Dahlberg, L. L., & Krug, E. G. (2002). Violence: A global public health problem. In E. Krug, L. L. Dahlberg, J. A. Mercy, A. B. Zwi, & R. Lozano (Eds.), *World report on violence and health* (pp. 1–21). World Health Organization.

[CR22] Denk-Florea, C. B., Gancz, B., Gomoiu, A., Ingram, M., Moreton, R., & Pollick, F. (2020). Understanding and supporting law enforcement professionals working with distressing material: Findings from a qualitative study. *Plos One*, *15*(11), e0242808. 10.1371/journal.pone.0242808 33237979 10.1371/journal.pone.0242808 PMC7688122

[CR23] Dennard, S., Tracy, D. K., Beeney, A., Craster, L., Bailey, F., Baureek, A., Barton, M., Turrell, J., Poynton, S., Navkarov, V., & Kothari, R. (2021). Working in a prison: Challenges, rewards, and the impact on mental health and well-being. *The Journal of Forensic Practice*, *23*(2), 132–149. 10.1108/JFP-12-2020-0055

[CR24] Drew, J. M., & Martin, S. (2021). A national study of police mental health in the USA: Stigma, mental health, and help-seeking behaviors. *Journal of Police and Criminal Psychology*, *36*(2), 295–306. 10.1007/s11896-020-09424-9

[CR25] Eades, N. D. (2020). Managing stressors in a detention facility: The need for supporting and safeguarding staff. *Journal of Adult Protection*, *22*(3), 153–163. 10.1108/JAP-12-2019-0040

[CR26] Edwards, K. L., Eaton-Stull, Y. M., & Kuehn, S. (2021). Police officer stress and coping in a stress-awareness era. *Police Quarterly*, *24*(3), 325–356. 10.1177/1098611120984162

[CR27] Ferdik, F., & Pica, E. (2023). Correctional officer turnover intentions and mental illness symptom: The potential confounding effects of resilience. *Psychology, Public Policy, and Law*. 10.1037/law0000384

[CR28] First Peoples Wellness Circle (2019). *The federal framework on PTSD act* [Briefing Note]. https://fpwc.ca/wp-content/uploads/2021/11/PTSD-FN-Briefing-Note.pdf

[CR29] Fortune, N., Rooney, B., & Kirwan, G. H. (2018). Supporting law enforcement personnel working with distressing material online. *Cyberpsychology Behavior and Social Networking*, *21*(2), 138–143. 10.1089/cyber.2016.0715 29048939 10.1089/cyber.2016.0715

[CR30] Fusco, N., Ricciardelli, R., Jamshidi, L., Carleton, R. N., Barnim, N., Hilton, Z., & Groll, D. (2021). When our work hits home: Trauma and Mental disorders in Correctional officers and other Correctional workers. *Frontiers in Psychiatry*, *11*, 493391. 10.3389/fpsyt.2020.493391 33658946 10.3389/fpsyt.2020.493391 PMC7917131

[CR31] Giwa, S., Colvin, R. A., Ricciardelli, R., & Warren, A. P. (2022). Workplace experiences of lesbian and bisexual female police officers in the Royal Newfoundland Constabulary. *Women & Criminal Justice*, *32*(1–2), 93–110. 10.1080/08974454.2021.1962480

[CR32] Hadjistavropoulos, H. D., McCall, H. C., Thiessen, D. L., Huang, Z., Carleton, R. N., Dear, B. F., & Titov, N. (2021). Initial outcomes of transdiagnostic internet-delivered cognitive behavioral therapy tailored to public safety personnel: Longitudinal observational study. *Journal of Medical Internet Research*, *23*(5), e27610. 10.2196/27610 33949959 10.2196/27610 PMC8135031

[CR33] Hean, S., Willumsen, E., & Ødegård, A. (2017). Collaborative practices between correctional and mental health services in Norway: Expanding the roles and responsibility competence domain. *Journal of Interprofessional care*, *31*(1), 18–27. 10.1080/13561820.2016.1233392 27918842 10.1080/13561820.2016.1233392

[CR34] Heber, A., Testa, V., Smith-MacDonald, L., Brémault-Phillips, S., & Carleton, R. N. (2020). Commentary — Rapid response to COVID-19: Addressing challenges and increasing the mental readiness of public safety personnel. *Health Promotion and Chronic Disease Prevention in Canada: Research Policy and Practice*, *40*(11–12), 350–355. 10.24095/hpcdp.40.11/12.04 32909935 10.24095/hpcdp.40.11/12.04 PMC7745835

[CR35] Hilton, Z., Ham, E., Rodrigues, N., Kirsh, B., Chapovalov, O., & Seto, M. (2020). *Appendix A: A research and knowledge translation project recommendations and suggested actions *[Knowledge translation document]. https://cdnsm5-hosted.civiclive.com/UserFiles/Servers/Server_9960/File/Research/Recommendations.pdf

[CR36] Hilton, N. Z., Addison, S., Ham, E., Rodrigues, C., N., & Seto, M. C. (2022). Workplace violence and risk factors for PTSD among psychiatric nurses: Systematic review and directions for future research and practice. *Journal of Psychiatric and Mental Health Nursing*, *29*(2), 186–203. 10.1111/jpm.12781 34214247 10.1111/jpm.12781

[CR37] Hilton, N. Z., Ricciardelli, R., Shewmake, J., Rodrigues, N. C., Seto, M. C., & Ham, E. (2021). Perceptions of Workplace Violence and Workplace Stress: A Mixed Methods Study of Trauma among Psychiatric Workers. *Issues in mental health nursing,**42*(9), 797–807. 10.1080/01612840.2021.1899350 33835903 10.1080/01612840.2021.1899350

[CR38] Housefather, A. (2018). *Improving support for jurors in Canada: Report of the standing committee on justice and human rights *[Report]. House of Commons Canada. https://www.ourcommons.ca/Content/Committee/421/JUST/Reports/RP9871696/justrp20/justrp20-e.pdf

[CR39] International Association of Chiefs of Police (2018a). *Officer safety and wellness: Practices in modern policing *[Report]. Alexandria, VA: International Association of Chiefs of Police. https://www.theiacp.org/sites/default/files/2018-11/IACP_PMP_SafetyandWellness.pdf

[CR40] Jaegers, L. A., Ahmad, S. O., Scheetz, G., Bixler, E., Nadimpalli, S., Barnidge, E., Katz, I. M., Vaughn, M. G., & Matthieu, M. M. (2020). Total worker health^®^ needs assessment to identify workplace mental health interventions in rural and urban jails. *The American Journal of Occupational Therapy*, *74*(3). 10.5014/ajot.2019.036400 10.5014/ajot.2019.036400 PMC7198237 32365308

[CR41] Jessiman-Perreault, G., Smith, M. P., & Gignac, M. A. M. (2021). Why are workplace social support programs not improving the mental health of Canadian correctional officers? An examination of the theoretical concepts underpinning support. *Environmental Research and Public Health*, *18*(2665), 1–11. 10.3390/ijerph18052665 10.3390/ijerph18052665 PMC7967375 33800869

[CR42] Johnston, M. S., Ricciardelli, R., & McKendy, L. (2022). Improving the mental health of correctional workers: Perspectives from the field. *Criminal Justice and Behavior*, *49*(7), 951–970. 10.21428/cb6ab371.55040b84

[CR43] Johnston, M. S., Ricciardelli, R., Ghodrati, M., & Czarnuch, S. (2023). Assessing road to mental readiness (R2MR) training among correctional workers in Canada. *Health & Justice*, *11*(2), 1–10. 10.1186/s40352-023-00206-z 36683119 10.1186/s40352-023-00206-z PMC9868502

[CR44] Martinez, V. R. (2019). Combating silence in the profession. *Virginia Law Review,**105*(4), 805–63. JSTOR, https://www.jstor.org/stable/26842255. Accessed 4 Apr 2024 .

[CR45] McCall, H. C., Beahm, J. D., Fournier, A. K., Burnett, J. L., Carleton, R. N., & Hadjistavropoulos, H. D. (2021a). Stakeholder perspectives on internet-delivered cognitive behavioural therapy for public safety personnel: A qualitative analysis. *Canadian Journal of Behavioural Science / Revue canadienne des Sciences du Comportement*, *53*(3), 232–242. 10.1037/cbs0000242

[CR46] McCall, H. C., Landry, C. A., Ogunade, A., Carleton, R. N., & Hadjistavropoulos, H. D. (2021b). Why do public safety personnel seek tailored internet-delivered cognitive behavioural therapy? An observational study of treatment-seekers. *International Journal of Environmental Research and Public Health*, *18*(22), 11972. 10.3390/ijerph182211972 34831728 10.3390/ijerph182211972 PMC8619750

[CR47] McCarty, W. P., Aldirawi, H., Dewald, S., & Palacios, M. (2019). Burnout in blue: An analysis of the extent and primary predictors of burnout among law enforcement officers in the United States. *Police Quarterly*, *22*(3), 278–304. 10.1177/1098611119828038

[CR48] Mental Health Commission of Canada (2017). *Case study research project findings *[Report]. https://www.mentalhealthcommission.ca/wp-content/uploads/drupal/2017-03/case_study_research_project_findings_2017_eng.pdf

[CR49] Mennicke, A., Gromer, J., Oehme, K., & MacConnie, L. (2018). Workplace experiences of gay and lesbian criminal justice officers in the United States: A qualitative investigation of officers attending a LGBT law enforcement conference. Policing and society, 28(6), 712-729.

[CR50] Miller, S. (2022). Moral injury, moral identity, and dirty hands in war fighting and police work. *Journal of Medicine and Philosophy: A Forum for Bioethics and Philosophy of Medicine*, *47*(6), 723–734. 10.1093/jmp/jhac028 10.1093/jmp/jhac028 36562840

[CR51] Moghimi, E., Knyahnytska, Y., Zhu, Y., Kumar, A., Knyahnytski, A., Patel, C., Omrani, M., Gerritsen, C., Martin, M., Simpson, F. I. A., & Alavi, N. (2022b). A qualitative exploration of the mental health challenges and therapeutic needs of Canadian correctional workers. *Frontiers in Psychiatry*, *13*, 1004143. 10.3389/fpsyt.2022.1004143 36386978 10.3389/fpsyt.2022.1004143 PMC9641701

[CR52] Munn, Z., Peters, M. D. J., Stern, C., Tufanaru, C., McArthur, A., & Aromataris, E. (2018). Systematic review or scoping review? Guidance for authors when choosing between a systematic or scoping review approach. *Bmc Medical Research Methodology*, *18*, 143. 10.1186/s12874-018-0611-x 30453902 10.1186/s12874-018-0611-x PMC6245623

[CR53] Murray, E. (2020). *Building police officer psychological capital to mitigate stress [Government Bulletin]*. FBI Law Enforcement Bulletin. https://leb.fbi.gov/articles/featured-articles/building-police-officer-psychological-capital-to-mitigate-stress

[CR54] National Officer Safety Initiatives (2018). *Preventing suicide among law enforcement officers: An issue brief*. International Association of Chiefs of Police. https://www.theiacp.org/sites/default/files/2020-02/_NOSI_Issue_Brief_FINAL.pdf

[CR55] National Union of Public and General Employees (2024). *Mental injury among justice workers [report*]. https://nupge.ca/wp-content/uploads/2019/08/Mental-Injury-among-Justice-Workers-Report-2024.pdf.

[CR56] Newell, C. J., Ricciardelli, R., Czarnuch, S. M., & Martin, K. (2022). Police staff and mental health: Barriers and recommendations for improving help-seeking. *Police Practice and Research*, *23*(1), 111–124. 10.1080/15614263.2021.1979398

[CR57] Oliphant, R. (2016). *Healthy minds, safe communities: Supporting our public safety officers through a national strategy for operational stress injuries*. House of Commons Canada.

[CR58] Papazoglou, K., Collins, P. I., Blumberg, D. M., Schlosser, M., & Bonanno, G. (2021). *Death and loss in law enforcement*. FBI Law Enforcement Bulletin. https://leb.fbi.gov/articles/featured-articles/death-and-loss-in-law-enforcement

[CR59] Pitel, M. C., Ewles, G. B., Hausdorf, P. A., & Heffren, C. D. (2021). Post-traumatic effects in policing: Exploring disclosure, coping, and social support. *Police Practice and Research*, *22*(1), 308–323. 10.1080/15614263.2020.1848564

[CR60] Police Executive Research Forum (2021). *Promising strategies for strengthening police department wellness programs: Findings and recommendations from the officer safety and wellness technical assistance project*. Office of Community Oriented Policing Services. https://portal.cops.usdoj.gov/resourcecenter/ric/Publications/cops-w0964-pub.pdf

[CR61] Public Safety Canada (2019). *Supporting Canada’s public safety personnel: An action plan on post-traumatic stress injuries*. https://www.publicsafety.gc.ca/cnt/rsrcs/pblctns/2019-ctn-pln-ptsi/index-en.aspx

[CR62] Ramchand, R., Saunders, J., Osilla, K. C., Ebener, P., Kotzias, V., Thornton, E., Strang, L., & Cahill, M. (2018). Suicide prevention in U.S. law enforcement agencies: A national survey of current practices. *Journal of Police and Criminal Psychology*, *34*(1), 55–66. 10.1007/s11896-018-9269-x

[CR63] Ricciardelli, R., Power, N., & Medeiros, D. S. (2018a). Correctional officers in Canada: Interpreting workplace violence. *Criminal Justice Review*, *43*(4), 458–476. 10.1177/0734016817752433

[CR64] Ricciardelli, R., Carleton, R. N., Mooney, T., & Cramm, H. (2018c). Playing the system: Structural factors potentiating mental health stigma, challenging awareness, and creating barriers to care for Canadian public safety personnel. *Health: An Interdisciplinary Journal for the Social Study of Health Illness and Medicine*, *24*(3), 259–278. 10.1177/1363459318800167 10.1177/1363459318800167 32283964

[CR65] Ricciardelli, R., Carleton, R. N., Gacek, J., & Groll, D. (2020a). Understanding needs, breaking down barriers: Examining mental health challenges and well-being of correctional staff in Ontario, Canada. *Frontiers in Psychology*, *11*, 1036. 10.3389/fpsyg.2020.01036 32754074 10.3389/fpsyg.2020.01036 PMC7365997

[CR66] Ricciardelli, R., Czarnuch, S., Carleton, R. N., Gacek, J., & Shewmake, J. (2020b). Canadian public safety personnel and occupational stressors: How PSP interpret stressors on duty. *International Journal of Environmental Research and Public Health*, *17*(13), 4736. 10.3390/ijerph17134736 32630259 10.3390/ijerph17134736 PMC7370189

[CR67] Ricciardelli, R., Cassiano, M. S., Adorjan, M., & Mitchell, M. M. (2021a). AMStrength program in Canadian federal correctional services: Correctional officers’ views and interpretations. *Criminal Justice Studies*, *34*(4), 459–476. 10.1080/1478601X.2021.1997277

[CR68] Ricciardelli, R., Haynes, S. H., Burdette, A., Keena, L., McCreary, D. R., Carleton, R. N., Lambert, E. G., & Groll, D. (2021b). Mental health stigma, gender, and seeking treatment: Interpretations and experiences of prison employees. *Applied Psychology in Criminal Justice*, *16*(1), 107–127.

[CR69] Ricciardelli, R., Norman, M., & Maier, K. (2022b). *The mental health and well-being of Canadian federal parole officers: A qualitative investigation*. https://usje-sesj.com/wp-content/uploads/2022/05/PO.REPORT.MAY9.2022.DIGITAL.pdf

[CR70] Ricciardelli, R., Mario, B., Sibley, A. M., & Johnston, S. M. (2023a). *Correctional worker experiences and ideas for future design at her Majesty’s Penitentiary*. The Newfoundland and Labrador Association of Public and Private Employees.

[CR71] Ricciardelli, R., Mitchell, M. M., Taillieu, T., Cassiano, M. S., Afifi, T. O., & Carleton, R. N. (2023b). Exposures to correctional-specific potentially psychologically traumatic events among Ontario provincial correctional workers. *Psychological Trauma: Theory Research Practice and Policy*, *15*(Suppl 2), S246–S258. 10.1037/tra0001437 36848056 10.1037/tra0001437

[CR72] Rodrigues, N. C., Ham, E., Hilton, N. Z., & Seto, M. C. (2021a). Workplace characteristics of forensic and nonforensic psychiatric units associated with posttraumatic stress disorder (PTSD) symptoms. *Psychological Services*, *18*(4), 464. 10.1037/ser0000405 31944816 10.1037/ser0000405

[CR73] Rodrigues, N. C., Ham, E., Kirsh, B., Seto, M. C., & Hilton, N. Z. (2021b). Mental health workers’ experiences of support and help-seeking following workplace violence: A qualitative study. *Nursing & Health Sciences*, *23*(2), 381–388. 10.1111/nhs.12816 33496379 10.1111/nhs.12816

[CR74] Rodriguez, S., Ferrell, B., CiprianoJr, R. J., Van Hasselt, V. B., Falzone, L., Kuhlman, K., Wesley, A., & Miller, M. V. (2023). Law enforcement mental health: Strategies and issues in prevention and treatment. *Practice Innovations*. 10.1037/pri0000210

[CR75] Sapers, H., Murphy, Y., Walker, C. M., Monteiro, A., Crete, J. P., & St-Cyr, K. (2018). Institutional violence in Ontario. *Final report*. Case study: Toronto South Detention Centre. Independent Review of Ontario Corrections. https://www.correctionsdivision.ca/wp-content/uploads/2019/03/2018-Violence-Report.pdf

[CR76] Seto, M. C., Rodrigues, N. C., Ham, E., Kirsh, B., & Hilton, N. Z. (2020). Post-traumatic stress disorder, Depression, anxiety symptoms, and help seeking in Psychiatric Staff. *Canadian Journal of Psychiatry*, *65*(8), 577–583. 10.1177/0706743720916356 32228305 10.1177/0706743720916356 PMC7492885

[CR77] Sewell, J. D. (2021a). *Developing a critical incident peer support program: Model policy *[Guidance Document]. Office of Community Oriented Policing Services. https://portal.cops.usdoj.gov/resourcecenter/RIC/Publications/cops-w0942-pub.pdf

[CR78] Sewell, J. D. (2021b). *Effective leadership response to the challenges of law enforcement suicide *[Guidance Document]. Office of Community Oriented Policing Services. https://portal.cops.usdoj.gov/resourcecenter/RIC/Publications/cops-w0944-pub.pdf

[CR79] Sewell, D. J. (2021c). *Guide for developing an effective stress management policy for law enforcement: Psychological support, training of agency personnel, cardiovascular disease, and police suicide *[Guidance Document]. Office of Community Oriented Policing Services. https://portal.cops.usdoj.gov/resourcecenter/RIC/Publications/cops-w0943-pub.pdf

[CR80] Siqueira Cassiano, M., Ricciardelli, R., & Foley, G. (2022). The mental health and wellness of correctional officers in Canada: Programs and practices. *Corrections*, 1–18. 10.1080/23774657.2022.2052380

[CR81] Stelnicki, A. M., Jamshidi, L., Fletcher, A. J., & Carleton, R. N. (2021). Evaluation of before operational stress: A program to support mental health and proactive psychological protection in public safety personnel. *Frontiers in Psychology*, *12*, 3218. 10.3389/fpsyg.2021.511755 10.3389/fpsyg.2021.511755 PMC8416101 34484013

[CR82] Supreme Judicial Court Standing Committee on Lawyer Well-Being & Massachusetts Bar Association (2021). *The path to lawyer well-being: A toolkit for bar associations in Massachusetts* [Guidance Document]. Massachusetts Bar Association. https://www.massbar.org/docs/default-source/wellbeing/mbawellbeingtoolkit.pdf

[CR83] Swenson, D., Bibelhausen, J., Buchanan, B., Shaheed, D., & Yetter, K. (2020). Stress and resiliency in the US Judiciary. *Journal of the Professional Lawyer*. https://www.courts.michigan.gov/499539/siteassets/committees-boards-special-initiatves/lawyer-well-being/stress-and-resiliency-in-the-us-judiciary.pdf

[CR84] Task Force on Wisconsin Lawyer Well-Being (2021). *Lawyer well-being: Changing the climate of Wisconsin’s legal profession* [Report]. State of Wisconsin Bar. https://www.wisbar.org/NewsPublications/Documents/Lawyer%20well-being%20-%20changing%20the%20climate%20of%20wisconsins%20legal%20profession%20-%20dec%202021%20-%20bog%20report.pdf

[CR85] Taylor, M. A. (2022). Building resilience in law enforcement through a mental wellness program. *Journal of Police and Criminal Psychology*, *37*(1), 155–161. 10.1007/s11896-021-09479-2

[CR86] Taylor, B. G., Maitra, P., Mumford, E., & Liu, W. (2022). Sexual harassment of law enforcement officers: Findings from a nationally representative survey. *Journal of Interpersonal Violence*, *37*(11–12). 10.1177/0886260520978180. NP8454-NP8478. 10.1177/0886260520978180 33283599

[CR87] Tricco, A. C., Lillie, E., Zarin, W., O’Brien, K. K., Colquhoun, H., Levac, D., Moher, D., Peters, M. D. J., Horsley, T., Weeks, L., Hempel, S., Akl, E. A., Chang, C., McGowan, J., Stewart, L., Hartling, L., Aldcroft, A., Wilson, M. G., Garritty, C., & Straus, S. E. (2018). PRISMA Extension for scoping reviews (PRISMA-ScR): Checklist and Explanation. *Annals of Internal Medicine*, *169*(7), 467–473. 10.7326/M18-0850 30178033 10.7326/M18-0850

[CR88] Union of Safety and Justice Employees (2019). *Protecting public safety: The challenges facing federal parole officers in Canada’s highly stressed criminal justice system* [Report]. https://s3.amazonaws.com/usge-web/uploads/docs/USJE+Parole+Officer+Report_EN.pdf

[CR89] Union of Solicitor General Employees (2017). *Moving forward: A report on the invisible toll of psychological trauma on federal public safety workers* [Report]. https://usje-sesj.com/wp-content/uploads/2021/07/movingforward.pdf

[CR90] Vanhove, A. J., Herian, M. N., Perez, A. L. U., Harms, P. D., & Lester, P. B. (2016). Can resilience be developed at work? A meta-analytic review of resilience-building programmeeffectiveness. *Journal of Occupational and Organizational Psychology,**89*(2), 278–307. 10.1111/joop.12123

[CR91] Veritas Health Innovation (2023). *Covidence systematic review software*. Melbourne, Australia. Available at www.covidence.org

[CR92] Violanti, J. M. (2017). *Building resilience: A protective leadership strategy for increasing performance *[Guidance Document]. The Police Chief.

[CR93] Wild, J., El-Salahi, S., & Degli Esposti, M. (2020a). The effectiveness of interventions aimed at improving well-being and resilience to stress in first responders: A systematic review. *European Psychologist*, *25*(4), 252–271. 10.1027/1016-9040/a000402

[CR94] Wild, J., El-Salahi, S., Degli Esposti, M., & Thew, G. R. (2020b). Evaluating the effectiveness of a group-based resilience intervention versus psychoeducation for emergency responders in England: A randomised controlled trial. *PloS One*, *15*(11), e0241704. 10.1371/journal.pone.0241704 33180798 10.1371/journal.pone.0241704 PMC7660584

[CR95] WorkSafeBC (2024). *Mental health presumption extended to 11 new occupations*. https://www.worksafebc.com/en/about-us/news-events/announcements/2024/June/mental-health-presumption-extended-to-11-new-occupations

[CR96] WorkSafeNB (2023). *Feedback sought on proposed legislative change for correctional officers making PTSD claims*. https://www.worksafenb.ca/about-us/news-and-events/news/2023/feedback-sought-on-proposed-legislative-change-for-correctional-officers-making-ptsd-claims/

[CR97] Yu, H. H. (2017). Post-executive order 13583: A reexamination of occupational barriers in federal law enforcement. *Women & Criminal Justice*, *27*(4), 205–218. 10.1080/08974454.2016.1256253

[CR98] Zeng, X., Zhang, X., Chen, M., Liu, J., & Wu, C. (2020). The influence of perceived organizational support on police job burnout: a moderated mediation model. *Frontiers in Psychology*, *11*, 948. 10.3389/fpsyg.2020.00948 32528368 10.3389/fpsyg.2020.00948 PMC7265159

