# Supplementary material for: Psychological health and safety of criminal justice workers: a scoping review of strategies and supporting research
Source: Health Justice. 2025 Feb 26;13:10. doi: 10.1186/s40352-025-00320-0 (PMC11863410; doi:10.1186/s40352-025-00320-0)
Supplement: Supplementary file 1 — Supplementary Material 1. [file 40352_2025_320_MOESM1_ESM.docx]

**Supplemental Table 1: Definitions of Terminology**

| **Term** | **Definition** |
| --- | --- |
| **Framework, strategies, guidelines** | Government or organizational documents and reports that outline plans to achieve future goals or make recommendations for determining future action. |
| **Guidelines** | A recommended course of action that guides or directs action in a situation. A guideline allows for flexibility to use professional judgement, such that compliance is not mandatory. Also defined as empirically supported practices that focus on specific practice questions and client situations. |
| **Model** | A structure that outlines a set of beliefs and assumptions, and organizes knowledge and skills, to delineate the scope and methods of practice. Also categorized as two distinct systems that medical professionals use in clinical work and research. |

**Supplemental Table 2: Criteria for inclusion and exclusion in the scoping review by population**

| **Included populations** | **Excluded populations** |
| --- | --- |
| Populations included in this scoping review were individuals who work within, or those whose work directly relates to, the criminal justice system, including:   - - Law enforcement personnel   - Lawyers (defense counsel and prosecutors), paralegals/law clerks, and assistants/judges   - Other staff from courts (e.g., court clerks, court reporters)   - All staff in correctional institutions (e.g., officers, guards, wardens, administrative staff)   - Correctional healthcare staff (e.g., nurses, doctors, psychologists, psychiatrists)   - Peer support workers and groups   - Jurors   - Justice workers and case   - Managers   - Individuals supporting the transition from corrections to community   - Probation and parole officers   - Mobile crisis teams   - Staff in forensic institutions (e.g., nurses, doctors, psychologists, psychiatrics)   - Forensic administrative staff (management, senior leaders)   - NGOs   - Private practitioners   - Social workers   - Legal counsel who practice in mental health and criminal law | Populations excluded from this study were:   - Justice-involved individuals (e.g., individuals who have interacted with police, been accused, charged, and/or convicted of a crime, individuals who are incarcerated, etc.), inclusive of youth and adults - Victims of crime - Others impacted by crime (e.g., children and other family members) |

**Supplemental Table 3: Sample search strategy: EBSCO search platform**

| **#** | **Query** | **Results** |
| --- | --- | --- |
| S13 | S4 AND S8 AND S11  **Limiters:**  Published Date: 2017-2023  Language: English  Publication: Academic Journals  **Expanders:** Equivalent Subjects  **Search modes:** Boolean/Phrase | **3,943** |
| S12 | S4 AND S8 AND S11 | 11,697 |
| S11 | S9 OR S10 | 9,694,614 |
| S10 | AB Framework* OR Standard* OR Guideline* OR Policy OR Policies OR “Best Practice” OR Strategy* OR Recommendation* OR barrier* OR challenge* | 9,052,872 |
| S9 | TI “scoping review” OR “systematic review” OR meta-analysis OR synthesis OR “rapid review” OR “umbrella review” OR “narrative review” | 925,645 |
| S8 | S5 OR S6 OR S7 | 2,632,050 |
| S7 | Occupational OR Employee OR Workplace OR Work N2 (“Health and Safety” OR Health OR Safety) | 1,115,854 |
| S6 | SU Trauma OR “Compassion Fatigue” OR “Mental Health” OR “Mental Illness” OR Stress OR Burnout | 1,642,753 |
| S5 | “Psychological Health and Safety” OR “Psychological Safety” | 2,373 |
| S4 | S1 OR S2 OR S3 | 115,712 |
| S3 | (Prison OR Jail OR Correction* OR Police OR Probation or Parole OR “Law enforcement” OR Law OR Legal OR Justice or Court OR Forensic OR “Public Safety”) N2 (Officer* OR Worker* OR Personnel OR Guard* OR Reporter* OR Staff OR Employee* OR Nurse* OR Psychiatrist* OR Psychologist* OR advocate* OR “Peer Support”) | 56,184 |
| S2 | (Police OR Warden* OR Lawyer* OR Attorney OR Prosecutor* OR Paralegal* OR “Law Clerk”* OR Juror* OR Jury OR “First Responder” OR “Emergency Responder” OR “Criminal Justice” OR “Gladue Writer* OR “Criminal Justice System”) | 120,362 |
| S1 | SU Judges or judiciary or judicial | 7,923 |

**Supplemental Table 4: Records included in the scoping review**

| **Citation** | **Type** | **Population** | **Jurisdiction** |
| --- | --- | --- | --- |
| [Alrutz, A. S., Buetow, S., Cameron, L. D., & Huggard, P. K. (2020). What Happens at Work Comes Home. Healthcare (Basel, Switzerland), 8(3), 350. https://doi.org/10.3390/healthcare8030350](https://doi.org/10.3390/healthcare8030350) | Academic | Police officers/Law enforcement personnel | New Zealand |
| [Anderson, G. S., Di Nota, P. M., Groll, D., & Carleton, R. N. (2020). Peer Support and Crisis-Focused Psychological Interventions Designed to Mitigate Post-Traumatic Stress Injuries among Public Safety and Frontline Healthcare Personnel: A Systematic Review. International journal of environmental research and public health, 17(20), 7645. https://doi.org/10.3390/ijerph17207645](https://doi.org/10.3390/ijerph17207645) | Academic | Correctional officers; Police officers/Law enforcement personnel; Forensic and correctional nurses | Canada; USA; Australia; New Zealand; United Kingdom |
| Arter, M. L., & Menard, K.S. (2018). An examination of the reasons police officers fail to seek treatment for occupational stress. Law Enforcement Executive Forum, 18(1), 30-42. | Academic | Police officers/Law enforcement personnel | USA |
| [Auditor General of Canada. (2017). Report 4 - Mental health support for members - Royal Canadian Mounted Police [Audit Report]. Parliament of Canada. https://www.oag-bvg.gc.ca/internet/English/parl_oag_201705_04_e_42226.html](https://www.oag-bvg.gc.ca/internet/English/parl_oag_201705_04_e_42226.html) | Grey | Police officers/Law enforcement personnel | Canada |
| Banwell-Moore, R., Tomczak, P., Wainwright, L., Traynor, C., & Hyde, S. (2022). 'The human toll': Highlighting the unacknowledged harms of prison suicide which radiate across stakeholder groups. Incarceration, 3(2), 1-20. DOI:10.1177/26326663221097337 | Academic | Correctional officers; Correctional administrative staff; Forensic and correctional nurses; Forensic and correctional psychiatrists; Forensic and correctional psychologists; Peer support workers; Probation and parole officers; Mental health court workers/mental health court diversion workers | Canada; USA; New Zealand; United Kingdom |
| [Bastarache, M. (2021). Broken dreams broken lives: The devastating effects of sexual harassment on women in the RCMP. A final report on the implementation of the Merlo Davidson settlement agreement. https://www.rcmp-grc.gc.ca/wam/media/4773/original/8032a32ad5dd014db5b135ce3753934d.pdf](https://www.rcmp-grc.gc.ca/wam/media/4773/original/8032a32ad5dd014db5b135ce3753934d.pdf) | Grey | Police officers/Law enforcement personnel | Canada |
| Benuto, L. T., Singer, J., Gonzalez, F., Newlands, R., & Hooft, S. (2019). Supporting those who provide support: Work-related resources and secondary traumatic stress among victim advocates. Safety and health at work, 10(3), 336-340. | Academic | Victim advocates | USA |
| Beshai, S., Carelton, R. N., Dirkse, A. D., Duranceau, A., Hamptom, D. J. A., Ivens, E. S., LeBouthillier, M. D., Tamaian, A., Sapach Teale, N. J. M., Thorisdottir, S. A., Walker, L. K., & Wuerch, A. M. (2016). Peer support and crisis-focused psychological intervention programs in Canadian first responders: Blue paper. | Grey | Police officers/Law enforcement personnel | Canada |
| [Beyond Blue Ltd. (2018). Answering the call national survey, National Mental Health and Wellbeing Study of Police and Emergency Services – Final report. https://www.beyondblue.org.au/docs/default-source/resources/bl1898-pes-full-report_final.pdf](https://www.beyondblue.org.au/docs/default-source/resources/bl1898-pes-full-report_final.pdf) | Grey | Police officers/Law enforcement personnel | Australia |
| [Beyond Blue. (2020). Good practice framework for mental health and wellbeing in police and emergency services organizations. Mind. https://www.beyondblue.org.au/docs/default-source/resources/bl2042_goodpracticeframework_a4.pdf](https://www.beyondblue.org.au/docs/default-source/resources/bl2042_goodpracticeframework_a4.pdf) | Grey | Police officers/Law enforcement personnel | Australia |
| Bouchard, L., Williams, D., Kiser, L., Freese, E., & Taren, D. (2022). Promoting professional quality of life and resiliency in sexual assault nurse examiners. Journal of Forensic Nursing, 18(1), 13 - 20. DOI: 10.1097/JFN.0000000000000350 | Academic | Forensic and correctional nurses | USA |
| [Boyle, A., Inger, A., Miller, M., & Waters, K. (2020). Breaking barriers, changing lives. Emergency Services Foundation. https://esf.com.au/wp-content/uploads/2020/05/ESF-Finalized-Booklet-SM.pdf](https://esf.com.au/wp-content/uploads/2020/05/ESF-Finalized-Booklet-SM.pdf) | Grey | Police officers/Law enforcement personnel | Australia |
| [Brafford, M. A. (2018). Well-being toolkit for lawyers and legal employers. The American Bar Association. https://lawyerwellbeing.net/wp-content/uploads/2020/02/Toolkit-Full_Final_July-30-2018.pdf](https://lawyerwellbeing.net/wp-content/uploads/2020/02/Toolkit-Full_Final_July-30-2018.pdf) | Grey | Lawyers | USA |
| [Brandhorst, K. J., & Compton, A. C. (2022). Constructing barriers to employee assistance program use by federal correctional officers. Journal of Applied Communication Research, 50(5), 497-514. https://doi.org/10.1080/00909882.2022.2032269](https://doi.org/10.1080/00909882.2022.2032269) | Academic | Correctional officers | USA |
| Buchanan, B., & Coyle, C. J. (2017). National task force on lawyer well-being: Creating a movement to improve well-being in the legal profession. American Bar Association. 1-73. | Grey | Lawyers; Judges | USA |
| Burdett, F., Gouliquer, L., & Poulin, C. (2018). Culture of corrections: The experiences of women correctional officers. Feminist Criminology, 13(3), 329-349. DOI: 10.1177/1557085118767974 | Academic | Correctional officers | Canada |
| Burgess, M. (2018). Inquiry into the role of commonwealth, state and territory Governments in addressing the high rates of mental health conditions experienced by first  responders, emergency service workers and volunteers. Police Federation of Australia. | Grey | Police officers/Law enforcement personnel | Australia |
| Cadieux, N., Cadieux, J., Youssef, N., Gingues, M., & Godbout, S.-M. (2020). Research  Report: A Study of the Determinants of Mental Health in the Workplace Among Quebec Lawyers, Phase II - 2017-2019. Research Report, Université de Sherbrooke, Business School, 1 - 177. | Grey | Lawyers | Canada |
| Canada, K. E., Watson, A. C., & O’kelley, S. (2021). Utilizing crisis intervention teams in prison to improve officer knowledge, stigmatizing attitudes, and perception of response options. Criminal justice and behavior, 48(1), 10-31. | Academic | Correctional officers | USA |
| [Carleton, N. (2021). Collaborating to support the mental health of public safety personnel: The Canadian Institute for Public Safety Research and Treatment. Canadian Psychology, 62(2), 167-173. https://psycnet.apa.org/doi/10.1037/cap0000267](https://psycnet.apa.org/doi/10.1037/cap0000267) | Academic | Correctional officers; Correctional administrative staff; Police officers/Law enforcement personnel | Canada |
| Clements, A. J., Sharples, A., & Kinman, G. (2021). Identifying well-being challenges and solutions in the police service: A World Café approach. The Police Journal, 94(2), 81-101. | Academic | Police officers/Law enforcement personnel | United Kingdom |
| Cohen, M. I., McCormick, V. A., & Rich, B. (2019). Creating a culture of police officer wellness. Oxford University Press, 13(2), 213-229. doi:10.1093/police/paz001 | Academic | Police officers/Law enforcement personnel | Canada |
| [College of Policing. (2020). Blue Light Wellbeing Framework. https://www.oscarkilo.org.uk/media/653/download?inline](https://www.oscarkilo.org.uk/media/653/download?inline) | Grey | Police officers/Law enforcement personnel | United Kingdom |
| College of Policing. (2022). Transformation framework: A standard approach to underpin police force strategic transformation and business change through peer support. College of Policing United Kingdom. | Grey | Police officers/Law enforcement personnel | United Kingdom |
| [Coopie, C., Coopie, J., Drake, J., Joyce, N., Robinson, M-J., Smoot, S., Stephens, D., & Villaseñor, R. (2019). Law enforcement and mental health and wellness programs: Eleven case studies. Washington, DC: Office of Community Oriented Policing Services. https://portal.cops.usdoj.gov/resourcecenter/RIC/Publications/cops-p371-pub.pdf](https://portal.cops.usdoj.gov/resourcecenter/RIC/Publications/cops-p371-pub.pdf) | Grey | Police officers/Law enforcement personnel | USA |
| Copple, C. K., & James E. C. (2018). Risk management in law enforcement: Discussions on identifying and mitigating risk for officers, departments, and the public. Washington, DC: Office of Community Oriented Policing Services | Grey | Police officers/Law enforcement personnel | USA |
| COPS Office (Office of Community Oriented Policing Services). (2018). Officer Health and Organizational Wellness: Emerging Issues and Recommendations. Officer Safety and Wellness Group Meeting Summary. Washington, DC: Office of Community Oriented Policing Services. | Grey | Police officers/Law enforcement personnel | USA |
| [Corthésy-Blondin, L., Genest, C., Dargis, L., Bardon, C., & Mishara, L. B. (2022). Reducing the impacts of exposure to potentially traumatic events on the mental health of public safety personnel: A rapid systematic scoping review. American Psychological Association, 19(52), 80-94. https://psycnet.apa.org/doi/10.1037/ser0000572](https://psycnet.apa.org/doi/10.1037/ser0000572) | Academic | Police officers/Law enforcement personnel | USA; Australia; United Kingdom |
| Crowe, A., Averett, P., Bonner, H., & Franks, C. (2022). “Let them know it’s okay to get help”: Addressing the Mental Health Needs of Police Officers. Administration and Policy in Mental Health and Mental Health Services Research, 49(4), 613-622. | Academic | Police officers/Law enforcement personnel | USA |
| Denk-Florea, C. B., Gancz, B., Gomoiu, A., Ingram, M., Moreton, R., & Pollick, F. (2020). Understanding and supporting law enforcement professionals working with distressing material: Findings from a qualitative study. Plos one, 15(11), e0242808. | Academic | Police officers/Law enforcement personnel | United Kingdom |
| Dennard, S., Tracy, D. K., Beeney, A., Craster, L., Bailey, F., Baureek, A., ... & Kothari, R. (2021). Working in a prison: Challenges, rewards, and the impact on mental health and well-being. The Journal of Forensic Practice, 23(2), 132-149. | Academic | Correctional officers; Correctional administrative staff; Forensic and correctional nurses; Forensic and correctional psychologists; Probation and parole officers | United Kingdom |
| [Di Nota, P. M., Bahji, A., Groll, D., Carleton, R. N., &amp; Anderson, G. S. (2021). Proactive psychological programs designed to mitigate posttraumatic stress injuries among at-risk workers: A systematic review and meta-analysis. Systematic Reviews, 10(1). https://doi.org/10.1186/s13643-021-01677-7](https://doi.org/10.1186/s13643-021-01677-7) | Academic | Correctional officers; Police officers/Law enforcement personnel | Canada |
| Dixon, S. S. (2021). Law enforcement suicide: The depth of the problem and best practices for suicide prevention strategies. Aggression and violent behavior, 61, 101649. | Academic | Police officers/Law enforcement personnel | USA |
| Drew, J. M., & Martin, S. (2021). A national study of police mental health in the USA: stigma, mental health and help-seeking behaviors. Journal of police and criminal psychology, 36(2), 295-306. | Academic | Police officers/Law enforcement personnel | USA |
| [Eades, N. D. (2020). Managing stressors in a detention facility: The need for supporting and safeguarding staff. Journal of Adult Protection, 22(3), 153-163. https://doi.org/10.1108/JAP-12-2019-0040](https://doi.org/10.1108/JAP-12-2019-0040) | Academic | Correctional officers | Australia |
| Easterbrook, B., Ricciardelli, R., Sanger, B. D., Mitchell, M. M., McKinnon, M. C., & Carleton, R. N. (2022). Longitudinal study of Canadian correctional workers' wellbeing, organizations, roles and knowledge (CCWORK): Baseline demographics and prevalence of mental health disorders. Frontiers in Psychiatry, 13, 874997. | Academic | Correctional officers | Canada |
| [Edwards, A.-M., &amp; Kotera, Y. (2020). Mental health in the UK police force: A qualitative investigation into the stigma with mental illness. International Journal of Mental Health and Addiction, 19(4), 1116–1134. https://doi.org/10.1007/s11469-019-00214-x](https://doi.org/10.1007/s11469-019-00214-x) | Academic | Police officers/Law enforcement personnel | Canada; Australia; United Kingdom |
| Edwards, K. L., Eaton-Stull, Y. M., & Kuehn, S. (2021). Police officer stress and coping in a stress-awareness era. Police Quarterly, 24(3), 325-356. | Academic | Police officers/Law enforcement personnel | USA |
| [Ferdik, F., & Pica, E. (2023). Correctional Officer Turnover Intentions and Mental Illness Symptom: The Potential Confounding Effects of Resilience. Psychology, Public Policy, and Law. https://doi.org/10.1037/law0000384](https://doi.org/10.1037/law0000384) | Academic | Correctional officers | Canada; USA |
| Finney, C., Stergiopoulos, E., Hensel, J., Bonato, S., & Dewa, C. S. (2013). Organizational stressors associated with job stress and burnout in correctional officers: a systematic review. BMC public health, 13, 1-13. | Academic | Correctional officers | Canada; USA |
| [First Peoples Wellness Circle. (2019). The federal framework on PTSD act (Briefing Note). https://fpwc.ca/wp-content/uploads/2021/11/PTSD-FN-Briefing-Note.pdf](https://fpwc.ca/wp-content/uploads/2021/11/PTSD-FN-Briefing-Note.pdf) | Grey | Police officers/Law enforcement personnel | Canada |
| Fisher, M. P., & Lavender, C. D. (2023). Ensuring optimal mental health programs and policies for first responders: opportunities and challenges in one US state. Community mental health journal, 1-11. | Academic | Police officers/Law enforcement personnel | USA |
| Fletcher, A., McKie, L., MacPherson, I., & Tombs, J. (2021). Impacts of professionalization and wellbeing policies on Scottish prison workers. Frontiers in Sociology, 6, 757583. | Academic | Correctional officers | United Kingdom |
| Fortune, N., Rooney, B., & Kirwan, G. H. (2018). Supporting law enforcement personnel working with distressing material online. Cyberpsychology, Behavior, and Social Networking, 21(2), 138-143. | Academic | Police officers/Law enforcement personnel | USA |
| Future Thinking. (2016). Blue light programme: Stand 1 Part 2 [Evaluation Report]. Mind. | Grey | Police officers/Law enforcement personnel | United Kingdom |
| Giwa, S., Colvin, R. A., Ricciardelli, R., & Warren, A. P. (2022). Workplace experiences of lesbian and bisexual female police officers in the Royal Newfoundland Constabulary. Women & Criminal Justice, 32(1-2), 93-110. | Academic | Police officers/Law enforcement personnel | Canada |
| Hadjistavropoulos, H. D., McCall, H. C., Thiessen, D. L., Huang, Z., Carleton, R. N., Dear, B. F., & Titov, N. (2021). Initial outcomes of transdiagnostic internet-delivered cognitive behavioral therapy tailored to public safety personnel: longitudinal observational study. Journal of Medical Internet Research, 23(5), e27610. | Academic | Correctional officers; Police officers/Law enforcement personnel | Canada |
| [Hallinan, S., Shiyko, M., Volpe, R., & Molnar, B. E. (2021). On the back burner: Challenges experienced by change agents addressing vicarious trauma in first response and victim service agencies. Traumatology, 27(3), 316–325. https://doi.org/10.1037/trm0000291](https://doi.org/10.1037/trm0000291) | Academic | Police officers/Law enforcement personnel | USA |
| Heber, A., Testa, V., Smith-MacDonald, L., Brémault-Phillips, S., & Carleton, R. N. (2020). Commentary-Rapid response to COVID-19: Addressing challenges and increasing the mental readiness of public safety personnel. Health Promotion and Chronic Disease Prevention in Canada: Research, Policy and Practice, 40(11-12), 350. | Academic | Correctional officers; Police officers/Law enforcement personnel | Canada |
| [Hesketh, I., & Tehrani, N. (2017). Psychological risk management: Introduction and guidance. College of Policing. https://assets.college.police.uk/s3fs-public/2021-02/psychological-risk-management.pdf](https://assets.college.police.uk/s3fs-public/2021-02/psychological-risk-management.pdf) | Grey | Police officers/Law enforcement personnel | United Kingdom |
| [Hesketh, I., & Tehrani, N. (2018). Responding to trauma in policing: A practical guide. College of Policing. https://assets.college.police.uk/s3fs-public/2021-02/responding-to-trauma-in-policing.pdf](https://assets.college.police.uk/s3fs-public/2021-02/responding-to-trauma-in-policing.pdf) | Grey | Police officers/Law enforcement personnel | United Kingdom |
| [Hesketh, I., &amp; Tehrani, N. (2018). Psychological trauma risk management in the UK Police Service. Policing: A Journal of Policy and Practice, 13(4), 531–535. https://doi.org/10.1093/police/pay083](https://doi.org/10.1093/police/pay083) | Academic | Police officers/Law enforcement personnel | United Kingdom |
| Hilton, N. Z., Addison, S., Ham, E., C Rodrigues, N., & Seto, M. C. (2022). Workplace violence and risk factors for PTSD among psychiatric nurses: Systematic review and directions for future research and practice. Journal of Psychiatric and Mental Health Nursing, 29(2), 186-203. | Academic | Forensic and correctional nurses | Canada; USA; Australia; United Kingdom; Norway |
| Hilton, Z., Ham, E., Rodrigues, N., Kirsh B., Chapovalov, O., Seto, M. (2020) Appendix A: A Research and Knowledge Translation Project Recommendations and Suggested Actions. | Academic | Forensic and correctional nurses; Forensic administrative staff | Canada |
| Hofer, M. S., & Savell, S. M. (2021). “There was no plan in place to get us help”: Strategies for improving mental health service utilization among law enforcement. Journal of police and criminal psychology, 36, 543-557. | Academic | Police officers/Law enforcement personnel | USA |
| Horowitz, V. L., Greberman, E. R., Nolan, P. E., Hyatt, J. M., Uggen, C., Andersen, S. N., & Chanenson, S. L. (2021). A comparative perspective on officer wellness: american reflections from norwegian prisons. Criminal Justice Studies, 34(4), 477-497. | Academic | Correctional officers | USA |
| [Housefather, A. (2018). Improving support for jurors in Canada: Report of the standing committee on justice and human rights. House of Commons Canada. https://www.ourcommons.ca/Content/Committee/421/JUST/Reports/RP9871696/justrp20/justrp20-e.pdf](https://www.ourcommons.ca/Content/Committee/421/JUST/Reports/RP9871696/justrp20/justrp20-e.pdf) | Grey | Jurors | Canada |
| Huey, L., Norman, M., Ricciardelli, R., & Spencer, D. C. (2023). “I’ve Seen More Dead People than I thought I Would”: Vicarious Trauma Exposure among Police Support Personnel. Criminal Justice and Behavior, 50(4), 541-558. | Academic | Police officers/Law enforcement personnel | United Kingdom |
| [IACP. (2018). Suicide prevention, intervention, and postventtion: Policy guidance for law enforcement agencies. International Association of Chiefs of Police. https://www.theiacp.org/sites/default/files/Suicide%20Prevention%2C%20Intervention%2C%20%26%20Postvention%20Policy%20Guidance%20for%20Law%20Enforcement%20Agencies_0.pdf](https://www.theiacp.org/sites/default/files/Suicide%20Prevention%2C%20Intervention%2C%20%26%20Postvention%20Policy%20Guidance%20for%20Law%20Enforcement%20Agencies_0.pdf) | Grey | Police officers/Law enforcement personnel | USA |
| International Association of Chiefs of Police & United States of America. (2020). National Consortium on Preventing Law Enforcement Suicide Final Report. | Grey | Police officers/Law enforcement personnel | USA |
| International Association of Chiefs of Police. (2018). Officer Safety and Wellness. Practices in Modern Policing. Alexandria, VA: International Association of Chiefs of Police. https://www.theiacp.org/sites/default/files/2018-11/IACP_PMP_SafetyandWellness.pdf | Grey | Police officers/Law enforcement personnel | USA |
| International Association of Chiefs of Police. (2018). Officer safety and wellness: Practices in modern policing. Alexandria, VA: International Association of Chiefs of Police | Grey | Police officers/Law enforcement personnel | USA |
| [International Association of Chiefs of Police. (2023). Implementing Peer Support Services in Small and Rural Law Enforcement Agencies. Washington, DC: Office of Community Oriented Policing Services. https://portal.cops.usdoj.gov/resourcecenter/RIC/Publications/cops-w0987-pub.pdf](https://portal.cops.usdoj.gov/resourcecenter/RIC/Publications/cops-w0987-pub.pdf) | Grey | Police officers/Law enforcement personnel | USA |
| [Jaegers, L. A., Ahmad, S. O., Scheetz, G., Bixler, E., Nadimpalli, S., Barnidge, E., Katz, I. M., Vaughn, M. G., &amp; Matthieu, M. M. (2020). Total worker health® needs assessment to identify workplace mental health interventions in rural and urban jails. The American Journal of Occupational Therapy, 74(3). https://doi.org/10.5014/ajot.2019.036400](https://doi.org/10.5014/ajot.2019.036400) | Academic | Correctional officers | USA |
| Jamalulhak, A. I., Galletly, C., & Ford, N. (2022). Improving services for those who serve: A private practice initiative to improve psychiatric care for first responder and military patients. Australasian psychiatry, 30(3), 308-312. | Academic | Police officers/Law enforcement personnel | Australia |
| [Jessiman-Perreault, G., Smith, M. P., & Gignac, M. A. M. (2021). Why are workplace social support programs not improving the mental health of Canadian correctional officers? An examination of the theoretical concepts underpinning support. Environmental Research and Public Health, 18(2665), 1-11. https://doi.org/10.3390/ijerph18052665](https://doi.org/10.3390/ijerph18052665) | Academic | Correctional officers | Canada |
| [Johnston, M. S., Ricciardelli, R., &amp; McKendy, L. (2022). Improving the mental health of Correctional Workers: Perspectives from the field. CrimRxiv. https://doi.org/10.21428/cb6ab371.55040b84](https://doi.org/10.21428/cb6ab371.55040b84) | Academic | Correctional officers | Canada |
| [Johnston, M. S., Ricciardelli, R., Ghodrati, M., & Czarnuch, S. (2023). Assessing Road to Mental Readiness (R2MR) training among correctional workers in Canada. Health & Justice, 11(1), 2. https://doi.org/10.1186/s40352-023-00206-z](https://doi.org/10.1186/s40352-023-00206-z) | Academic | Correctional officers | Canada |
| Jones, C., Bright, K., Smith-MacDonald, L., Pike, A. D., & Bremault-Phillips, S. (2022). Peers supporting reintegration after occupational stress injuries: A qualitative analysis of a workplace reintegration facilitator training program developed by municipal police for public safety personnel. The Police Journal, 95(1), 152-169. | Academic | Correctional officers; Police officers/Law enforcement personnel | Canada |
| Jones, C., Smith-MacDonald, L., Pike, A., Bright, K., & Bremault-Phillips, S. (2022). Workplace reintegration facilitator training program for mental health literacy and workplace attitudes of public safety personnel: pre-post pilot cohort study. JMIR formative research, 6(4), e34394. | Academic | Police officers/Law enforcement personnel | Canada |
| Kelk, N., Luscombe, G., Medlow, S., & Hickie, I. (2009). Courting the blues: Attitudes towards depression in Australian law students and lawyers. Brain & Mind Research Institute: University of Sydney. | Grey | Lawyers | Australia |
| [Kendall, C. (2011). Report on psychological distress and depression in the legal profession. Law Society of Western Australia. https://nla.gov.au/nla.obj-504224610/view](https://nla.gov.au/nla.obj-504224610/view) | Grey | Lawyers | Australia |
| [Konyk, K.; Ricciardelli, R.; Taillieu, T.; Afifi, T.O.; Groll, D.; Carleton, R.N. Assessing Relative Stressors and Mental Disorders among Canadian Provincial Correctional Workers. Int. J. Environ. Res. Public Health 2021, 18, 10018. https://doi.org/10.3390/ijerph181910018](https://doi.org/10.3390/ijerph181910018) | Academic | Correctional officers | Canada |
| LaMontagne, A. D., Martin, A. J., Page, K. M., Papas, A., Reavley, N. J., Noblet, A. J., ... & Smith, P. M. (2021). A cluster RCT to improve workplace mental health in a policing context: Findings of a mixed‐methods implementation evaluation. American Journal of Industrial Medicine, 64(4), 283-295. | Academic | Police officers/Law enforcement personnel | Australia |
| [Law Care. (2021). Life in the law. https://www.lawcare.org.uk/media/14vhquzz/lawcare-lifeinthelaw-v6-final.pdf](https://www.lawcare.org.uk/media/14vhquzz/lawcare-lifeinthelaw-v6-final.pdf) | Grey | Lawyers; Paralegals/Law Clerks and Assistants | United Kingdom |
| [Law Enforcement Mental Health and Wellness Act of 2017, United States of America. https://www.govinfo.gov/content/pkg/COMPS-13866/pdf/COMPS-13866.pdf](https://www.govinfo.gov/content/pkg/COMPS-13866/pdf/COMPS-13866.pdf) | Grey | Police officers/Law enforcement personnel | USA |
| Lawrence-Wood, E., Sadler, N., O’Dwyer, C., & Dell, L. (2021) Understanding stigma and help-seeking in emergency services personnel for good mental health. Report prepared for Emergency Services Foundation.  Phoenix Australia – Centre for Posttraumatic Mental Health: Melbourne. | Grey | Police officers/Law enforcement personnel | Australia |
| McCall, H. C., Beahm, J. D., Fournier, A. K., Burnett, J. L., Carleton, R. N., & Hadjistavropoulos, H. D. (2021). Stakeholder perspectives on internet-delivered cognitive behavioural therapy for public safety personnel: A qualitative analysis. Canadian Journal of Behavioural Science/Revue canadienne des sciences du comportement, 53(3), 232. | Academic | Correctional officers; Police officers/Law enforcement personnel | Canada |
| McCall, H. C., Landry, C. A., Ogunade, A., Carleton, R. N., & Hadjistavropoulos, H. D. (2021). Why do public safety personnel seek tailored internet-delivered cognitive behavioural therapy? An observational study of treatment-seekers. International journal of environmental research and public health, 18(22), 11972. | Academic | Correctional officers; Police officers/Law enforcement personnel | Canada |
| McCarty, W. P., Aldirawi, H., Dewald, S., & Palacios, M. (2019). Burnout in blue: an analysis of the extent and primary predictors of burnout among law enforcement officers in the United States. Police quarterly, 22(3), 278-304. | Academic | Police officers/Law enforcement personnel | USA |
| Mental Health Commission of Canada (2017). Case Study Research Project Findings. Ottawa, ON: Mental Health Commission of Canada. | Grey | Police officers/Law enforcement personnel; Forensic and correctional nurses; Forensic and correctional psychiatrists; Forensic and correctional psychologists; Forensic administrative staff; Social workers in forensic settings; Psychometrists | Canada |
| Michalak, T. R.(2015). Causes and consequences of work-related psychosocial risk exposure: A comparative investigation of organizational context, employee attitudes, job performance and wellbeing in lawyers and non-lawyer professionals. ISBN: 978-0-9874106-0-3 (Print version) ISBN: 978-0-9874106-1-0 (Electronic version) | Grey | Lawyers; Paralegals/Law Clerks and Assistants | Australia; New Zealand |
| Miller, S. (2022, December). Moral Injury, Moral Identity, and “Dirty Hands” in War Fighting and Police Work. In The Journal of Medicine and Philosophy: A Forum for Bioethics and Philosophy of Medicine (Vol. 47, No. 6, pp. 723-734). US: Oxford University Press. | Academic | Police officers/Law enforcement personnel | Australia; United Kingdom |
| Milliard, B. (2020). Utilization and impact of peer-support programs on police officers’ mental health. Frontiers in psychology, 1686. | Academic | Police officers/Law enforcement personnel | Canada |
| Moghimi, E., Knyahnytska, Y., Omrani, M., Nikjoo, N., Stephenson, C., Layzell, G., ... & Alavi, N. (2022). Benefits of digital mental health care interventions for correctional workers and other public safety personnel: a narrative review. Frontiers in Psychiatry, 13, 921527. | Academic | Correctional officers | Canada; USA; United Kingdom |
| Moghimi, E., Knyahnytska, Y., Zhu, Y., Kumar, A., Knyahnytski, A., Patel, C., ... & Alavi, N. (2022). A qualitative exploration of the mental health challenges and therapeutic needs of Canadian correctional workers. Frontiers in Psychiatry, 13, 1004143. | Academic | Correctional officers | Canada |
| Murray, E. (2020). Building Police Officer Psychological Capital to Mitigate Stress. FBI Law Enforcement Bulletin. | Grey | Police officers/Law enforcement personnel | USA |
| National Alliance on Mental Illness. (2016). Preparing for the Unimaginable: How Chiefs Can Safeguard Officer Mental Health Before and After Mass Casualty Events. Washington, DC: Office of Community Oriented Policing Services. | Grey | Police officers/Law enforcement personnel | USA |
| [National Consortium on Preventing Law Enforcement Suicide. (2018). Comprehensive framework for law enforcement suicide prevention. Bureau of Justice Assistance. https://www.theiacp.org/sites/default/files/2021-09/_NOSI_Framework_Final%20Copy%2001.pdf](https://www.theiacp.org/sites/default/files/2021-09/_NOSI_Framework_Final%20Copy%2001.pdf) | Grey | Police officers/Law enforcement personnel | USA |
| [National Officer Safety Initiatives. (2018). Preventing suicide among law enforcement officers: An issue brief. International Association of Chiefs of Police. https://www.theiacp.org/sites/default/files/2020-02/_NOSI_Issue_Brief_FINAL.pdf](https://www.theiacp.org/sites/default/files/2020-02/_NOSI_Issue_Brief_FINAL.pdf) | Grey | Police officers/Law enforcement personnel | USA |
| National Union of Public and General Employees. (2019). Mental injury among justice workers. | Grey | Correctional officers; Correctional administrative staff; Police officers/Law enforcement personnel | Canada |
| Newell, C. J., Ricciardelli, R., Czarnuch, S. M., & Martin, K. (2022). Police staff and mental health: Barriers and recommendations for improving help-seeking. Police practice and research, 23(1), 111-124. | Academic | Police officers/Law enforcement personnel | Canada |
| Oates, J., Topping, A., Ezhova, I., Wadey, E., & Rafferty, A. M. (2021). Factors affecting high secure forensic mental health nursing workforce sustainability: Perspectives from frontline nurses and stakeholders. Journal of psychiatric and mental health nursing, 28(6), 1041-1051. | Academic | Forensic and correctional nurses; Forensic administrative staff | United Kingdom |
| Oliphant, R. (2016). Healthy minds, safe communities: Supporting our public safety officers through a national strategy for operational stress injuries. House of Commons Canada. | Grey | Correctional officers; Police officers/Law enforcement personnel; Probation and parole officers | Canada |
| Papazoglou, K., Bonanno, G., Blumberg, D., & Keesee, T. (2019). Moral injury in police work. FBI Law Enforcement Bulletin, 9-20. | Grey | Police officers/Law enforcement personnel | Canada; USA |
| Papazoglou, K., Collins, P. I., Blumberg, D. M., Schlosser, M., & Bonanno, G. (2021). Death and Loss in Law Enforcement. | Grey | Police officers/Law enforcement personnel | USA |
| Pitel, M. C., Ewles, G. B., Hausdorf, P. A., & Heffren, C. D. (2021). Post-traumatic effects in policing: exploring disclosure, coping and social support. Police Practice and Research, 22(1), 308-323. | Academic | Police officers/Law enforcement personnel | Canada |
| [Police Executive Research Forum. (2021). Promising Strategies for Strengthening Police Department Wellness Programs: Findings and recommendations from the officer safety and wellness technical assistance project. Washington, DC: Office of Community Oriented Policing Services. https://portal.cops.usdoj.gov/resourcecenter/ric/Publications/cops-w0964-pub.pdf](https://portal.cops.usdoj.gov/resourcecenter/ric/Publications/cops-w0964-pub.pdf) | Grey | Police officers/Law enforcement personnel | USA |
| [Police Federation of Australia. (2021). Inquiry into mental health and suicide prevention. https://pfa.org.au/wp-content/uploads/2021/08/PFA-PC-Mental-Health-submission-FINAL.pdf](https://pfa.org.au/wp-content/uploads/2021/08/PFA-PC-Mental-Health-submission-FINAL.pdf) | Grey | Police officers/Law enforcement personnel | Australia |
| [Police Federation. (n.d.). Stress in the police service. Supporting mental wellbeing in the workplace: Our commitment. https://www.polfed.org/media/14122/tackling-stress-in-the-police-service-published-9-point-plan-template.pdf](https://www.polfed.org/media/14122/tackling-stress-in-the-police-service-published-9-point-plan-template.pdf) | Grey | Police officers/Law enforcement personnel | United Kingdom |
| [Public Safety Canada. (2019). Supporting Canada's Public Safety Personnel: An action plan on post-traumatic stress injuries. https://www.publicsafety.gc.ca/cnt/rsrcs/pblctns/2019-ctn-pln-ptsi/index-en.aspx](https://www.publicsafety.gc.ca/cnt/rsrcs/pblctns/2019-ctn-pln-ptsi/index-en.aspx) | Grey | Correctional officers; Police officers/Law enforcement personnel | Canada |
| [Ramchand, R., Saunders, J., Osilla, K. C., Ebener, P., Kotzias, V., Thornton, E., Strang, L., &amp; Cahill, M. (2018). Suicide prevention in U.S. law enforcement agencies: A national survey of current practices. Journal of Police and Criminal Psychology, 34(1), 55–66. https://doi.org/10.1007/s11896-018-9269-x](https://doi.org/10.1007/s11896-018-9269-x) | Academic | Police officers/Law enforcement personnel | USA |
| Redmond, T., Conway, P., Bailey, S., Lee, P., & Lundrigan, S. (2023). How we can protect the protectors: learning from police officers and staff involved in child sexual abuse and exploitation investigations. Frontiers in Psychology, 14, 1152446. | Academic | Police officers/Law enforcement personnel | United Kingdom |
| Ricciardelli, R. R., Haynes, S. H., Burdette, A., Keena, L., McCreary, D. R., Carleton, R. N., ... & Groll, D. (2021). Mental health stigma, gender and seeking treatment: Interpretations and experiences of prison employees. Applied Psychology in Criminal Justice, 16(1), 107-127. | Academic | Correctional officers | Canada |
| [Ricciardelli, R., Carleton, R. N., Gacek, J., & Groll, L. D. (2020). Understanding needs, breaking down barriers: Examining mental health challenges and well-being of correctional staff in Ontario, Canada. Frontiers in Psychology, 11(1036), 1-10. https://doi.org/10.3389/fpsyg.2020.01036](https://doi.org/10.3389/fpsyg.2020.01036) | Academic | Correctional officers | Canada |
| Ricciardelli, R., Cassiano, M. S., Adorjan, M., & Mitchell, M. M. (2021). AMStrength program in Canadian federal correctional services: Correctional officers’ views and interpretations. Criminal Justice Studies, 34(4), 459-476. | Academic | Correctional officers | Canada |
| Ricciardelli, R., Czarnuch, S., Carleton, R. N., Gacek, J., & Shewmake, J. (2020). Canadian public safety personnel and occupational stressors: How PSP interpret stressors on duty. International Journal of Environmental Research and Public Health, 17(13), 4736. | Academic | Correctional officers; Police officers/Law enforcement personnel | Canada |
| [Ricciardelli, R., Huey, L., Norman, M., & Spencer, C. D. (2022). The mental health and well-being of detachment services assistants in the Royal Canadian Mounted Police: A qualitative investigation. Union of Safety and Justice Employees. https://usje-sesj.com/wp-content/uploads/2022/03/RCMP.DSA_.REPORT.03.22.pdf](https://usje-sesj.com/wp-content/uploads/2022/03/RCMP.DSA_.REPORT.03.22.pdf) | Grey | Police officers/Law enforcement personnel | Canada |
| Ricciardelli, R., Mario, B., Sibley, A. M., & Johnston, S. M. (2023). Correctional worker experiences and ideas for future design at Her [His] Majesty's Penitentiary. The Newfoundland and Labrador Association of Public and Private Employees. | Grey | Correctional officers | Canada |
| Ricciardelli, R., Mitchell, M. M., Taillieu, T., Cassiano, M. S., Afifi, T. O., & Carleton, R. N. (2023). Exposures to correctional-specific potentially psychologically traumatic events among Ontario provincial correctional workers. Psychological trauma: theory, research, practice, and policy. | Academic | Correctional officers; Correctional administrative staff | Canada |
| [Ricciardelli, R., Norman, M., Maier, K. (2022). The mental health and well-being of Canadian federal parole officers: A qualitative investigation. https://usje-sesj.com/wp-content/uploads/2022/05/PO.REPORT.MAY9_.2022.DIGITAL.pdf](https://usje-sesj.com/wp-content/uploads/2022/05/PO.REPORT.MAY9_.2022.DIGITAL.pdf) | Grey | Probation and parole officers | Canada |
| Ricciardelli, R., Power, N., & Medeiros, D. S. (2018). Correctional officers in Canada: Interpreting workplace violence. Criminal Justice Review, 43(4), 458-476. | Academic | Correctional officers | Canada |
| [Robertson, E. W. (2019). Implementation of a standardized screening protocol to improve post-traumatic stress disorder surveillance in first responders. Journal of Occupational &amp;amp; Environmental Medicine, 61(12), 1041–1044. https://doi.org/10.1097/jom.0000000000001732](https://doi.org/10.1097/jom.0000000000001732) | Academic | Police officers/Law enforcement personnel | USA |
| Rodrigues, N. C., Ham, E., Hilton, N. Z., & Seto, M. C. (2021). Workplace characteristics of forensic and nonforensic psychiatric units associated with posttraumatic stress disorder (PTSD) symptoms. Psychological Services, 18(4), 464. | Academic | Forensic and correctional nurses; Forensic and correctional psychiatrists; Forensic and correctional psychologists; Social workers in forensic settings; Psychometrists | Canada |
| Rodrigues, N. C., Ham, E., Kirsh, B., Seto, M. C., & Hilton, N. Z. (2021). Mental health workers' experiences of support and help‐seeking following workplace violence: A qualitative study. Nursing & Health Sciences, 23(2), 381-388. | Academic | Correctional officers | Canada |
| Rodriguez, S., Ferrell, B., Cipriano Jr, R. J., Van Hasselt, V. B., Falzone, L., Kuhlman, K., ... & Miller, M. V. (2023). Law enforcement mental health: Strategies and issues in prevention and treatment. Practice Innovations. | Academic | Police officers/Law enforcement personnel | USA |
| [Sapers, H., Murphy, Y., Walker, C. M., Monteiro, A., Crete, J. P., & St-Cyr, K. (2018). Institutional violence in Ontario. Final report. Case study: Toronto South Detention Centre. Independent Review of Ontario Corrections. Retrieved from https://www. correctionsdivision. ca/wpcontent/uploads/2019/03/2018-Violence-Report. pdf.](https://www/) | Grey | Correctional officers; Correctional administrative staff | Canada |
| Sewell, D. J. (2021). Developing a critical incident peer support program: Model policy. Washington, DC: Office of  Community Oriented Policing Services. | Grey | Police officers/Law enforcement personnel | USA |
| Sewell, D. J. (2021). Guide for developing an effective stress management policy for law enforcement: Psychological  support, training of agency personnel, cardiovascular disease, and police suicide. Washington, DC: Office of Community Oriented Policing Services. | Grey | Police officers/Law enforcement personnel | USA |
| Sewell, J. (2021). Effective Leadership Response to the Challenges of Law Enforcement Suicide. | Grey | Police officers/Law enforcement personnel | USA |
| [Shields, R. (2018). Police mental health. A discussion paper. https://www.camh.ca/-/media/files/pdfs---public-policy-submissions/police-mental-health-discussion-paper-oct2018-pdf.pdf](https://www.camh.ca/-/media/files/pdfs---public-policy-submissions/police-mental-health-discussion-paper-oct2018-pdf.pdf) | Grey | Police officers/Law enforcement personnel | Canada |
| Siqueira Cassiano, M., Ricciardelli, R., & Foley, G. (2022). The Mental Health and Wellness of Correctional Officers in Canada: Programs and Practices. Corrections, 1-18. | Academic | Correctional officers | Canada |
| Slovinsky, T., & Brubaker, S. J. (2022). Prosecution as a “Soul Crushing” Job: Emotional Labor and Secondary Trauma in Working Sexual Assault Cases. Violence and victims. | Academic | Lawyers | USA |
| Spence, D. & Drake, J. (2021). Law Enforcement Suicide: 2020 Report to Congress. Washington, DC: U.S. Department of Justice. | Grey | Police officers/Law enforcement personnel | USA |
| Spence, D. (2017). Improving Law Enforcement Resilience: Lessons and  Recommendations. Officer Safety and Wellness Group Meeting Summary. Washington, DC: Office of Community Oriented Policing Services. | Grey | Police officers/Law enforcement personnel | USA |
| Spence, D., Fox, M., Moore, G., Estill, S., & Comrie, N. (2019). Law Enforcement Mental Health and Wellness Act: Report to Congress. Washington, DC: U.S. Department of Justice. | Grey | Police officers/Law enforcement personnel | USA |
| Stelnicki, A. M., Jamshidi, L., Fletcher, A. J., & Carleton, R. N. (2021). Evaluation of before operational stress: a program to support mental health and proactive psychological protection in public safety personnel. Frontiers in psychology, 12, 3218. | Academic | Correctional officers; Police officers/Law enforcement personnel | Canada |
| Stephenson, A. L., & Bell, N. (2019). Finding meaningful work in difficult circumstances: A study of prison healthcare workers. Health Services Management Research, 32(2), 69-77. | Academic | Forensic and correctional nurses | USA |
| [Supreme Judicial Court Standing Committee on Lawyer Well-Being & Massachusetts Bar Association. (2021). The path to lawyer well-being: A toolkit for bar associations in Massachusetts. Massachusetts Bar Association. https://www.massbar.org/docs/default-source/wellbeing/mbawellbeingtoolkit.pdf](https://www.massbar.org/docs/default-source/wellbeing/mbawellbeingtoolkit.pdf) | Grey | Lawyers | USA |
| Swenson, D., Bibelhausen, J., Buchanan, B., Shaheed, D., & Yetter, K. (2020). Stress and Resiliency in the US Judiciary. J. Prof. Law., 1. | Academic | Judges | USA |
| [Task Force on Wisconsin Lawyer Well-Being. (2021). Lawyer well-being: Changing the climate of Wisconsin's legal profession. State of Wisconsin Bar. https://www.wisbar.org/NewsPublications/Documents/Lawyer%20well-being%20-%20changing%20the%20climate%20of%20wisconsins%20legal%20profession%20-%20dec%202021%20-%20bog%20report.pdf](https://www.wisbar.org/NewsPublications/Documents/Lawyer%20well-being%20-%20changing%20the%20climate%20of%20wisconsins%20legal%20profession%20-%20dec%202021%20-%20bog%20report.pdf) | Grey | Lawyers; Paralegals/Law Clerks and Assistants | Canada |
| [Task Force on Wisconsin Lawyer Well-Being. (2021). Lawyer well-being: Changing the climate of Wisconsin's legal profession. State of Wisconsin Bar. https://www.wisbar.org/NewsPublications/Documents/Lawyer%20well-being%20-%20changing%20the%20climate%20of%20wisconsins%20legal%20profession%20-%20dec%202021%20-%20bog%20report.pdf](https://www.wisbar.org/NewsPublications/Documents/Lawyer%20well-being%20-%20changing%20the%20climate%20of%20wisconsins%20legal%20profession%20-%20dec%202021%20-%20bog%20report.pdf) | Grey | Lawyers; Judges | USA |
| Tatebe, L. C., Siva, N. R., Pekarek, S., Liesen, E., Wheeler, A., Reese, C., ... & Dennis, A. (2020). Heroes in crisis: Trauma centers should be screening for and intervening on posttraumatic stress in our emergency responders. Journal of trauma and acute care surgery, 89(1), 132-139. | Academic | Correctional officers; Police officers/Law enforcement personnel | USA |
| Taylor, B. G., Maitra, P., Mumford, E., & Liu, W. (2022). Sexual harassment of law enforcement officers: findings from a nationally representative survey. Journal of interpersonal violence, 37(11-12), NP8454-NP8478. | Academic | Police officers/Law enforcement personnel | USA |
| Taylor, M. A. (2022). Building resilience in law enforcement through a mental wellness program. Journal of Police and Criminal Psychology, 37(1), 155-161. | Academic | Police officers/Law enforcement personnel | USA |
| The International Association of Chiefs of Police Center for Officer Safety and Wellness. (2018) . The signs within: Suicide prevention education and awareness. Washington, DC: Office of Community Oriented Policing Services. | Grey | Police officers/Law enforcement personnel | USA |
| [The Police Journal: Theory, Practice and Principles. (2021). Organisational and individual perspectives of police wellbeing in England and Wales. 96(1), 128-152. https://doi.org/10.1177/0032258X211052250](https://doi.org/10.1177/0032258X211052250) | Academic | Police officers/Law enforcement personnel | United Kingdom |
| Union of Safety and Justice Employees (2019). Protecting Public Safety The challenges facing federal parole officers in Canada's highly stressed criminal justice system. | Grey | Probation and parole officers | Canada |
| Union of Solicitor General Employees (2017). Moving Forward: A Report on the Invisible Toll of Psychological Trauma on Federal Public Safety Workers. | Grey | Correctional officers; Correctional administrative staff; Police officers/Law enforcement personnel; Probation and parole officers | Canada |
| [United Nations Uniformed Capabilities Support Division. (2021). Comprehensive study to develop a PTSD framework for uniformed personnel. United Nations, Department of Operational Support. https://operationalsupport.un.org/sites/default/files/ptsd_study_final_report_with_appendix_0.pdf](https://operationalsupport.un.org/sites/default/files/ptsd_study_final_report_with_appendix_0.pdf) | Grey | Police officers/Law enforcement personnel; Mobile crisis response staff | Canada; USA; Australia; New Zealand; United Kingdom; Norway; Sweden; Denmark; Finland |
| Vickovic, S. G., Morrow, W. J., & Lambert, E. (2022). Examining the effects of job burnout and work-family conflict on correctional officer turnover intent. Criminal Justice Studies, 35(2), 111-131. | Academic | Correctional officers | USA |
| Violanti, J. M. (2017). Building resilience: a protective leadership strategy for increasing performance. The Police Chief. | Grey | Police officers/Law enforcement personnel | USA |
| Violanti, J. M., & Steege, A. (2020). Law enforcement worker suicide: an updated national assessment. Policing: An International Journal, 44(1), 18-31. | Academic | Police officers/Law enforcement personnel | USA |
| [Vision Research. (2012). Summary report: Survey of members in rural, remote and isolated communities. The Canadian Bar Association. https://www.cba.org/CBAMediaLibrary/cba_na/PDFs/CBA%20Wellness%20PDFs/lpac-communitysurvey2013-e.pdf](https://www.cba.org/CBAMediaLibrary/cba_na/PDFs/CBA%20Wellness%20PDFs/lpac-communitysurvey2013-e.pdf) | Grey | Lawyers; Judges | Canada |
| Voth, M., Chisholm, S., Sollid, H., Jones, C., Smith-MacDonald, L., & Brémault-Phillips, S. (2022). Efficacy, effectiveness, and quality of resilience-building mobile health apps for military, veteran, and public safety personnel populations: scoping literature review and app evaluation. JMIR mHealth and uHealth, 10(1), e26453. | Academic | Correctional officers; Police officers/Law enforcement personnel | Canada |
| Watson, L., & Andrews, L. (2018). The effect of a Trauma Risk Management (TRiM) program on stigma and barriers to help-seeking in the police. International journal of stress management, 25(4), 348. | Academic | Police officers/Law enforcement personnel | United Kingdom |
| Wild, J., El-Salahi, S., & Degli Esposti, M. (2020). The effectiveness of interventions aimed at improving well-being and resilience to stress in first responders. European Psychologist. | Academic | Police officers/Law enforcement personnel | USA; Australia; United Kingdom |
| Wild, J., El-Salahi, S., Degli Esposti, M., & Thew, G. R. (2020). Evaluating the effectiveness of a group-based resilience intervention versus psychoeducation for emergency responders in England: A randomised controlled trial. PloS one, 15(11), e0241704. | Academic | Police officers/Law enforcement personnel | United Kingdom |
| Wilson, S., Sinclair, A., Huxley, C., & Spiegelthalter, K. (2016). Evaluation of Mind’s Blue Light Programme. Strand 2: Workplace wellbeing. Institute for Employment Studies. Retrieved January, 11, 2020. | Grey | Police officers/Law enforcement personnel | United Kingdom |
| Yu, H. H. (2017). Post-executive order 13583: A reexamination of occupational barriers in federal law enforcement. Women & Criminal Justice, 27(4), 205-218. | Academic | Paralegals/Law Clerks and Assistants | USA |
